# Ocean Carbon Storage across the middle Miocene: a new interpretation for the Monterey Event

**DOI:** 10.1038/s41467-019-13792-0

**Published:** 2020-01-09

**Authors:** S. M. Sosdian, T. L. Babila, R. Greenop, G. L. Foster, C. H. Lear

**Affiliations:** 1 Cardiff University School of Earth and Ocean Sciences, Cardiff, CF10 3AT UK; 2 National Oceanography Centre Southampton, School of Ocean and Earth Science, University of Southampton, Waterfront Campus, Southampton, SO14 3ZA UK; 3 School of Earth and Environmental Science, University of St. Andrews, St. Andrews, KY16 9AL UK

**Keywords:** Biogeochemistry, Ocean sciences, Palaeoclimate

## Abstract

The Miocene Climatic Optimum (MCO, 14–17 Ma) was ~3–4 °C warmer than present, similar to estimates for 2100. Coincident with the MCO is the Monterey positive carbon isotope (δ^13^C) excursion, with oceans more depleted in ^12^C relative to ^13^C than any time in the past 50 Myrs. The long-standing Monterey Hypothesis uses this excursion to invoke massive marine organic carbon burial and draw-down of atmospheric CO_2_ as a cause for the subsequent Miocene Climate Transition and Antarctic glaciation. However, this hypothesis cannot explain the multi-Myr lag between the δ^13^C excursion and global cooling. We use planktic foraminiferal B/Ca, δ^11^B, δ^13^C, and Mg/Ca to reconstruct surface ocean carbonate chemistry and temperature. We propose that the MCO was associated with elevated oceanic dissolved inorganic carbon caused by volcanic degassing, global warming, and sea-level rise. A key negative feedback of this warm climate was the organic carbon burial on drowned continental shelves.

## Abstract (web summary)

In this study, the authors use planktic foraminiferal data to reconstruct ocean carbonate chemistry and temperature from 16.5 to 11 Ma from a size in the tropical eastern Indian Ocean to look at the causes of the Monterey Excursion (ME). They find a positive relationship between dissolved inorganic (DIC) carbon and the ME and a negative one for DIC and the carbon maxima events.

## Introduction

Understanding the causes of natural variations in atmospheric CO_2_ concentration requires an appreciation of the temporal distribution of carbon among the major reservoirs involved in carbon exchange on geological timescales. On long timescales, CO_2_ is added to the atmosphere via volcanic degassing, metamorphic decarbonation, and oxidation of organic matter, and is removed via chemical weathering and organic carbon burial^1,2^. The primary mechanisms that set this delicate balance of carbon inputs and outputs, which ultimately regulates long-term climate, are strongly debated^2–5^. Silicate weathering and organic carbon burial are proposed as key feedbacks in setting and/or mediating Neogene atmospheric CO_2_ levels^3,6,7^. Beyond understanding the geochemical carbon cycle, constraining the outcome of anthropogenic carbon addition requires an understanding of not only climate and ecological sensitivity to rapid carbon evasion^8,9^, but also the nature of different carbon cycle feedbacks. This latter requirement arises, because carbon cycle feedbacks can either enhance atmospheric CO_2_ trends or play a crucial part in their recovery. Numerous studies of Pleistocene climate records identified several carbon cycle feedback processes that operate or are enhanced when the Earth is in colder than modern climate modes^10,11^. In contrast, the feedback processes that operated during past warm climates are less well-studied and hence remain poorly understood, despite these being arguably more relevant for our warmer future. Carbon-cycle feedbacks are therefore currently not well represented in Earth System models and highlight a gap in our ability to predict future climate change^12,13^.

**Fig. 1:**
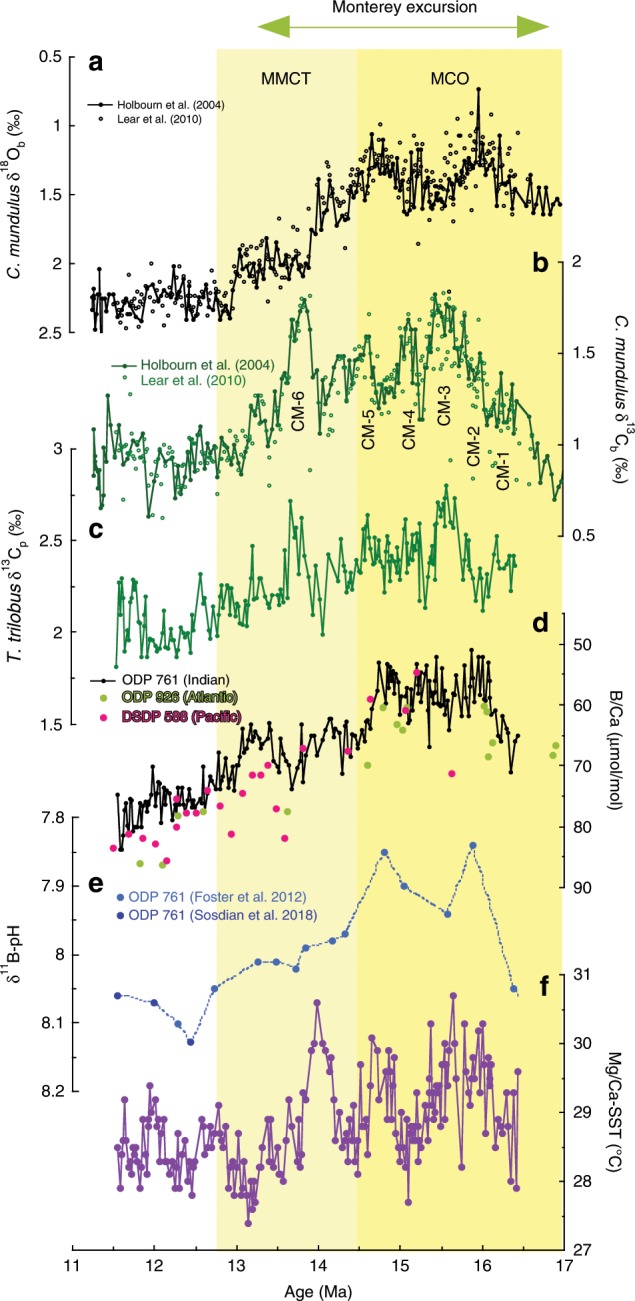
Isotopic and trace metal records across the middle Miocene. Previously published benthic foraminiferal (**a**) *C. mundulus* δ^18^O^14,21^ and (**b**) δ^13^C records from site ODP 761; (**c**) ODP 761 *T. trilobus* δ^13^C record from this study; (**d**) ODP 761 (Indian), DSDP 588 (Pacific), and ODP 926 (Atlantic) *T. trilobus* B/Ca µmol/mol, and (**e**) *T. trilobus* δ^11^B-pH estimates^29,35^ from site 761 for one δ^11^B_sw_ scenario^35,66^ (G17) with Monte Carlo uncertainty estimates (66% CI); (**f**) SST estimates derived from *T. trilobus* Mg/Ca at site 761. Yellow vertical bars highlight the timing of the Miocene Climatic Optimum (MCO) and Middle Miocene Climate Transition (MMCT). Carbon isotope maxima (CM) events 1–6 are highlighted. Monterey carbon isotope excursion duration is highlighted by the above arrow.

An ideal time interval to explore the controls on long-term atmospheric CO_2_ and pinpoint such carbon cycle feedbacks is the Miocene Climatic Optimum (MCO)—a period of sustained global warmth ~3 °C warmer than modern with reduced continental ice volume occurring ca. 14.7–17.0 Myrs ago and sitting within the Monterey carbon isotope excursion (MCIE)^14,15^. The MCIE was a prolonged (~3.5 Myrs) ~1.0 ‰ positive carbon isotope excursion (δ^13^C) of the global oceans, documented in planktic and benthic foraminifera records centered on ~15 Ma^16,17^ (Fig. 1), contemporaneous with basin-wide deposition of organic-rich deposits, as evident in the California Monterey Formation and elsewhere in the circum-Pacific Ocean^16,18,19^. The MCIE is usually described as comprising six carbon isotope maxima (CM events), which are paced by the ~400 kyr long-eccentricity cycle^17,20^ (Fig. 1b, c). Following the MCO, the middle Miocene Climate Transition (MMCT)ushered in a cooler and apparently more stable icehouse climate mode in a series of cooling or ice growth steps, as evidenced by benthic oxygen isotope records^17,21^(Fig. 1a) and a transition from a wet-based to dry-based Antarctic ice sheet^22^. This climate transition was associated with an overall decrease in the carbon isotopic composition of the ocean, although individual cooling steps have been associated with carbon maxima events, suggestive of the operation of positive carbon-cycle climate feedbacks^23^.

The classic interpretation, known as the Monterey Hypothesis^19^, proposed that long-term cooling increased the latitudinal gradient, invigorating upwelling, which led to increased organic carbon burial rates, lowering atmospheric CO_2_. This reinforced the initial cooling, while also increasing mean ocean δ^13^C composition and acting as a positive carbon cycle feedback. Subsequent hypotheses using strontium and osmium isotope records, invoked increased silicate weathering and enhanced nutrient delivery as a mechanism for CO_2_ draw down via enhanced organic carbon deposition and mid-Miocene cooling^6^. Others proposed the addition of volcanic CO_2_ emissions as a mechanism to maintain the MCO warmth^24,25^ and thereby stimulating high rates of chemical weathering alongside enhanced organic carbon burial. With the availability of the first continuous Miocene atmospheric CO_2_ records^26^ and identification of a minimum in CO_2_ concentrations during the MCO, it was hypothesized that the positive δ^13^C anomaly were caused by increased rates of organic carbon burial^19,26^ with the warmth of the MCO sustained by its particular oceanographic configuration and low continental ice volume rather than by greenhouse gas forcing^26^. However, these interpretations relied on limited and, in some cases, unreliable, available indices of middle Miocene atmospheric CO_2_, silicate/organic carbon weathering, and climate variability^6,19,24–27^. In particular, the early alkenone-based CO_2_ reconstructions were biased by diagenetic imprints on the original paleotemperature records^28^ that were used to calculate CO_2_.

Recent efforts to reconstruct CO_2_ using the boron isotope and alkenone-based paleobarometers, with more robust organic proxy-based temperature estimates, instead suggest that the warmth of the MCO was sustained by relatively high CO_2_ (470–630 ppm) and the MMCT was associated with falling CO_2_ concentrations^29–33^. It is now thought that CO_2_ emissions associated with the Columbia River Flood Basalts (CRB) may have been at least in part responsible for the elevated CO_2_ during the climatic optimum^24,29,31,32^. Furthermore, geochemical box modeling suggests that increased concentrations of CO_2_ may also account for part of the MCIE itself, by increasing the photosynthetic isotopic fraction of carbon to form organic matter more enriched in ^12^C on a global scale^25^. However, attributing the MCIE solely to a volcanic carbon source does not explain the ~400 kyr pacing of the carbon maxima events or the organic-rich sedimentation typified by the Monterey Formation and modeling produces a ~0.4‰ change in mean whole ocean δ^13^C, whereas the MCIE itself has a far greater amplitude (~1‰)^25^.

The association of the warmth of the MCO with elevated CO_2_ levels, organic-rich sedimentation, and positive carbon isotope excursions is reminiscent of the Cretaceous carbon cycle perturbations marked by widespread deposition of organic-rich sediments. This is thought to represent a key negative feedback in Earth’s ice-free greenhouse climate mode, helping to draw-down atmospheric CO_2_, but is one which came at a cost to Cretaceous marine ecosystems—widespread oceanic anoxia^33^. Although similar in many respects, the Miocene intervals of organic-rich sedimentation were never associated with widespread anoxia^34^. Records of ocean carbon chemistry are therefore required to understand the nature of the carbon cycle feedbacks in this warmer-than-present icehouse world.

Although the development of the boron isotope (δ^11^B) proxy has contributed to the recognition that climate and CO_2_ were coupled across the MCO and the MMCT^23,29,30,35^, pH is just one aspect of the ocean carbonate system. Directly reconstructing relative changes in dissolved inorganic carbon (DIC) content of seawater would allow us to more firmly quantify changes in oceanic carbon storage, which would enable the carbon cycle feedback processes associated with the MCO to be investigated.

Here we use planktic foraminiferal boron to calcium (B/Ca) ratios along with complementary published δ^11^B records to reconstruct shifts in the concentration of [B(OH)_4_^−^/DIC] and [B(OH)_4_^−^/HCO_3_^−^] and relative changes in surface ocean DIC across the early Miocene, MCO, and MMCT. We present a moderately high resolution (one sample every ~23 kyr) multi-proxy record of B/Ca, Mg/Ca, and carbon isotope (δ^13^C_p_) variability in the planktic foraminifer *Trilobatus trilobus* spanning 16.5 to 11.5 Ma from Ocean Drilling Program (ODP) Site 761 located in the tropical eastern Indian Ocean. We supplement this record with lower resolution *T*. *trilobus* B/Ca reconstructions from the Pacific (Deep Sea Drilling Program (DSDP) Site 588; ODP Site 872) and Atlantic Ocean basins (ODP Site 926). We then apply ad hoc calibrations, based on modern culture and core-top datasets, along with existing records of pH to approximate shifts in surface ocean $\left[ {{\mathrm{B(OH)}}_4^ - /{\mathrm{DIC}}} \right]$ and [B(OH)_4_^−^/HCO_3_^−^], and ultimately DIC (see Methods). We then pair these records of ocean carbon storage with planktic Mg/Ca and δ^13^C_p_ records and previously published benthic δ^18^O records to provide a novel perspective on the MCIE and drivers of the carbon cycle across the middle Miocene. Our records show a positive relationship between DIC and the broad Monterey δ^13^C excursion, but a negative relationship between DIC and δ^13^C for the individual CM events. This suggests that the MCIE is not the sum of the carbon maxima events as originally supposed by the Monterey Hypothesis and instead that these two features of the carbon isotope record reflect different carbon cycle processes. We suggest that the broad MCIE represents a negative feedback process akin to Cretaceous Oceanic Anoxic Events (OAEs), which operated in a warmer-than-present icehouse world while the positive carbon isotope excursions (CM events) within the Monterey carbon excursion likely reflect a positive carbon cycle feedback process associated with orbital scale variability in marine productivity.

## Results

### B/Ca record of *T. trilobus* from the mid-Miocene

ODP Site 761 *T. trilobus* B/Ca, Mg/Ca, and δ^13^C_p_ records span a 5-million-year time slice beginning from the onset of the MCO, through its main body and into the MMCT (Fig. 1). At the onset of the MCIE, the planktic δ^13^C_p_ record from Site 761 shows an overall increase, following the benthic δ^13^C record, with average MCO δ^13^C_p_ values of 2.5 ‰ decreasing to ~2.0 ‰ by 12 Ma (Fig. 1). Superimposed upon this background, long-term trend are the globally recognized CM events^14^ associated with glacial events and positive shifts in δ^18^O_b_ during the middle Miocene.

During the onset of the MCO, *T. trilobus* B/Ca ratios decreases from ~70 µmol/mol to a minimum of 59 ± 1 µmol/mol ( ± 2 SE; 14.7 to 16.0 Ma). This decrease in B/Ca ratios is associated with increasing tropical Indian Ocean temperatures by ~1.8 °C (Fig. 1). Over the course of the middle Miocene, *T. trilobus* B/Ca increases across the MMCT from the minimum values of the MCO to a maximum B/Ca value of 76 ± 1 µmol/mol (±2 SE; 13.5–11.5 Ma), an average increase of 17 µmol/mol. This long-term increase in B/Ca records occurs in tandem with a small long-term temperature decrease of ~0.7 °C. The long-term increase in B/Ca values through the MMCT bears a remarkable similarity to the benthic δ^18^O (δ^18^O_b_) records and is expressed in two steps with the first B/Ca increase occurring at 14.7 Ma, with B/Ca values reaching 66 ± 1 (±2 SE) μmol/mol, before the final increase at 13 Ma to ~76 μmol/mol.

**Fig. 2:**
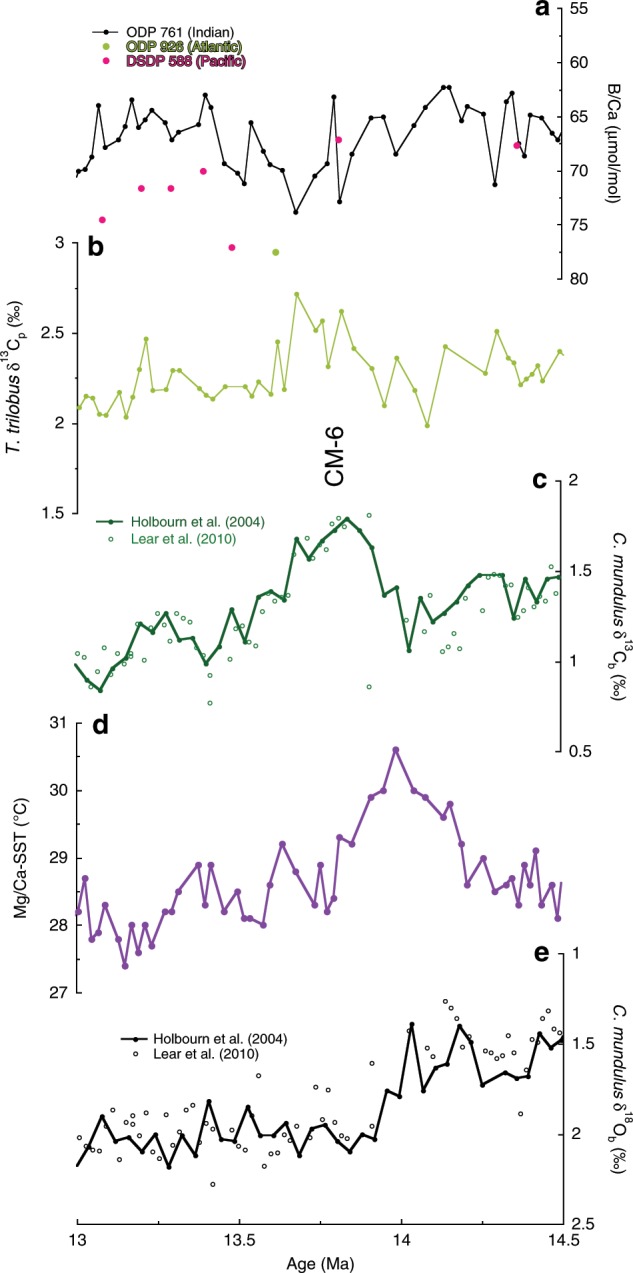
Isotopic and trace metal records across CM6. **a** Planktic foraminifera *T. trilobus* B/Ca from Indian (ODP 761), Pacific (DSDP 588), and Atlantic Oceans (ODP 926 alongside (**b**) planktic foraminiferal carbon isotope record from ODP site 761. **c** Benthic foraminiferal carbon isotopes from ODP site 761^14,21^. **d** Mg/Ca-SST estimates from ODP site 761. **e** Benthic foraminiferal oxygen isotopes from ODP site 761^14,21^.

Superimposed on this long-term B/Ca increase are numerous short-term variations in B/Ca with an amplitude between 10 and 15 µmol/mol. Earlier CM events show smaller scale peaks in B/Ca corresponding to the peak foraminiferal δ^13^C values. Peak positive planktic and benthic δ^13^C values during CM6 correspond to an increase B/Ca by ~10 μmol/mol. Further, the minimal long-term cooling in sea surface temperatures (SST) is punctuated by a sharp 2.4 °C cooling from 14.0 to 13.8 Ma, associated with the positive δ^18^O_b_ excursion and CM-6 event (Figs. 1 and 2). The overall SST amplitude at ODP 761 between warm, deglaciated and cool, glaciated conditions ranges from 3 °C to 2 °C, with a minimum temperature around 13.2 Ma.

We also present lower resolution *T. trilobus* B/Ca records from the Pacific (DSDP Site 588; ODP Site 872) and Atlantic (ODP Site 926) Ocean (Fig. 1 and Supplementary Figs. 1 and 2). These records show similarities in their general trends and absolute values to ODP Site 761, despite their different site locations, paleo water depths, and sediment burial depths. DSDP Site 588 B/Ca increases from 62 ± 4 µmol/mol (±2 SE) during the MCO to 80 ± 1 µmol/mol ( ± 2 SE), whereas ODP 926 increases from 65 ± 1 µmol/mol (±2 SE) during the MCO to 81 ± 2 µmol/mol ( ± 2 SE). This corresponds to a long-term increase in B/Ca of 18 and 16 µmol/mol in the Pacific and Atlantic, respectively, similar to Indian Ocean. ODP 872 B/Ca values decrease from the early Miocene into the MCO similar to the orbital-scale record from ODP 761, although our ODP 872 record is of lower resolution (Supplementary Fig. 2).

Overall, *T. trilobus* B/Ca records document a substantial decrease at the start of the MCIE, followed by an increase from the MCO through the MMCT in the Indian, Pacific, and Atlantic oceans. The similar trend and magnitude revealed by these B/Ca records, despite their diverse oceanographic settings suggests a global driver as the primary cause. In addition, the Site 761, Site 926, and Site 872 B/Ca records track the planktic δ^11^B-derived pH (i.e., MCO: low pH, low B/Ca) in the same cores supporting the idea that B/Ca is responding to global changes in surface ocean carbonate chemistry across the middle Miocene^29,30,35^ (Fig. 1 and Supplementary Fig. 2). Furthermore, the similar timing of change in the δ^13^C_p_ and B/Ca records across the middle Miocene suggests that the MCIE was associated with a change in the surface ocean carbon cycle and carbonate chemistry (Fig. 1).

The moderately resolved Indian Ocean *T. trilobus* B/Ca record shows a ~10 μmol/mol increase in B/Ca associated with CM6 (Fig. 2 and Supplementary Fig. 3). As the resolution of the Pacific and Atlantic B/Ca is too low to resolve short-term variations in surface ocean carbon storage, we look to the *T. trilobus* B/Ca record across CM6 from the Blue Clay Formation of Malta at Ras il-Pellegrin^23,36^ for comparison. This record shows an ~17 μmol/mol increase in B/Ca with a corresponding increase in planktic δ^11^B-derived pH. Together, the Indian Ocean and Malta records indicate surface ocean carbon reservoir changes associated with the short-term positive δ^13^C_p_ variations (i.e., CM events) in the global ocean (Supplementary Fig. 3).

### Reconstructing Ocean Carbon Storage across the middle Miocene

**Fig. 3:**
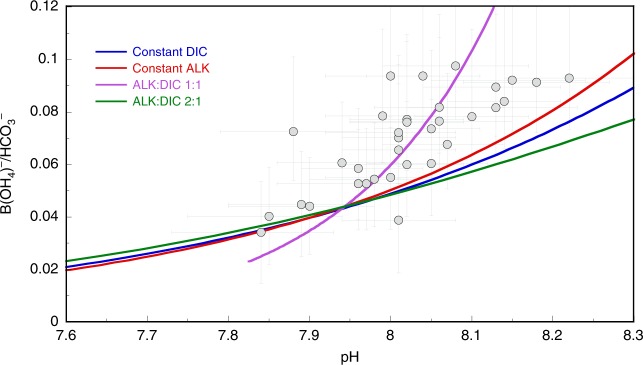
Sensitivity of pH vs. [B(OH)_4_^-^/HCO_3_^−^]_sw_ relationship to DIC and ALK. pH estimates derived from boron isotope reconstructions^35^ and ad hoc B/Ca-B(OH)_4_^−^/DIC and B/Ca-[B(OH)_4_^−^/HCO_3_^−^]_sw_ calibrations (see Methods) are plotted for a range of scenarios with changes in the dissolved inorganic carbon (DIC) and alkalinity (ALK) inventory. Blue and red line indicate scenarios with constant DIC and ALK, respectively. Purple and green lines indicate changes with a ALK:DIC ratio of 1:1 and 2:1. We plot one δ^11^B_sw_^35,66^ (G17) scenario to highlight the overall relationships present and provide full carbonate system estimates in the Methods. Monte Carlo uncertainty estimates (95% CI) are plotted for pH^35^ and [B(OH)_4_^−^/HCO_3_^−^]_sw_ estimates.

**Fig. 4:**
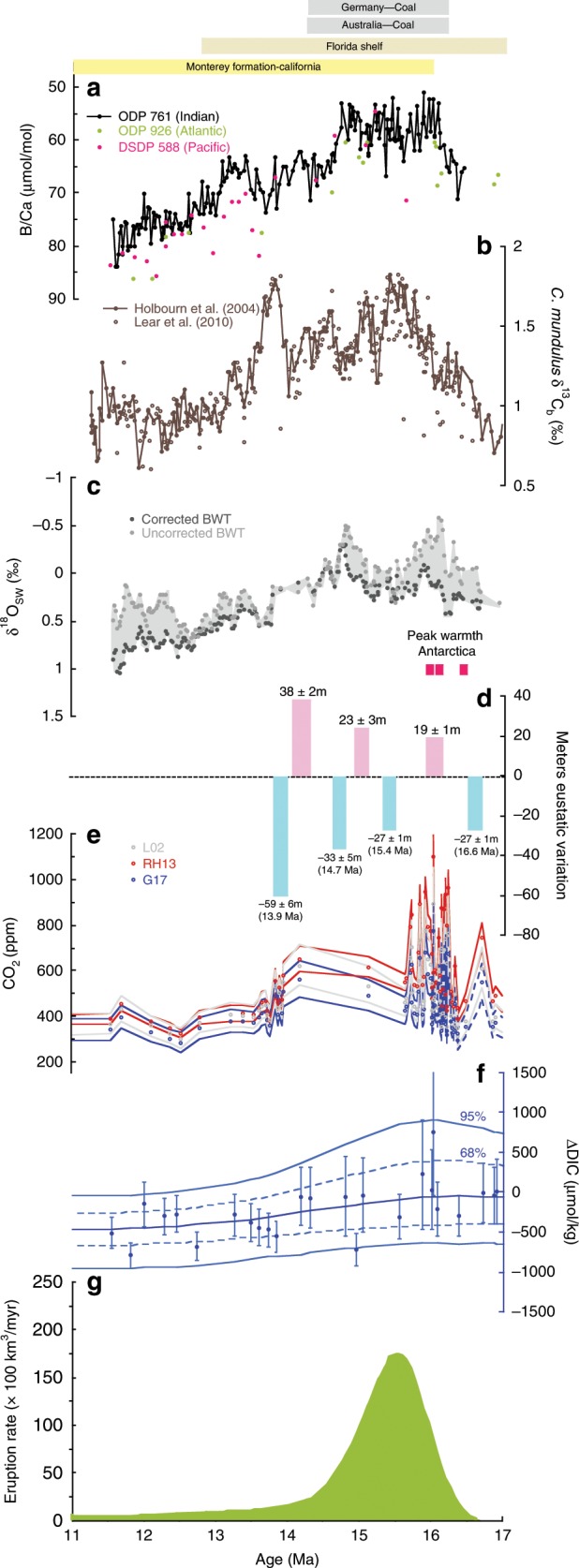
Paleoclimate records across the middle Miocene. **a** Planktic foraminifera *T. trilobus* B/Ca ratios from Indian (ODP 761), Pacific (DSDP 588), and Atlantic Oceans (ODP 926) alongside (**b**) benthic foraminiferal δ^13^C^14,21^ records from ODP site 761. **c** Estimates of changes in global ice volume as derived from δ^18^O_sw_ at ODP site 761. Gray circles are uncorrected BWT estimates, whereas dark gray circles are corrected^21^. **d** Eustatic sea-level change from the Marion Plateau^40^. Intervals of peak warmth in Antarctica as identified from ANDRILL^15^ are highlighted with pink bars (PW3-5). **e** Boron isotope derived atmospheric CO_2_ record^35^using three δ^11^B_sw_ scenarios^66,89,92^ (LO2; RH13; G17). **f** mid-Miocene DIC estimates with Monte Carlo uncertainty envelope using ad hoc B/Ca-B(OH)_4_^−^/DIC calibration. We plot one δ^11^B_sw_ scenario^35,66^ (G17) to highlight overall changes in DIC (△DIC) changes and provide full range of DIC estimates in the Methods. Miocene DIC values were subtracted from average MCO DIC levels to calculate △DIC. **g** Estimated eruption rate of the Columbia River Basalt^93^. Top bars specify marine (yellow) and land based (green) organic carbon deposition events evident across the Miocene^19,94,95^.

Using surface ocean pH estimates derived from boron isotope reconstructions^35^ and ad hoc B/Ca-B(OH)_4_^−^/DIC and B/Ca-B(OH)_4_^−^/HCO_3_^−^ calibrations (see Methods), we estimate changes in DIC and B(OH)_4_^−^/HCO_3_^−^ across the middle Miocene (Figs. 3 and 4).

First, this approach allows us to identify the likely drivers of ocean carbonate system change during the middle Miocene through examining the relationship between Miocene surface ocean B(OH)_4_^−^/HCO_3_^−^ and pH (Fig. 3). Changes in the ocean’s biological and carbonate pumps lead to alterations of the vertical gradients of DIC and alkalinity (ALK), but not their overall inventory. Whereas long-term shifts in carbon fluxes can alter ocean inventories of DIC and ALK with the stabilizing response of carbonate compensation processes (i.e., CaCO_3_ dissolution and formation). Specifically, if the change in the carbonate system reconstructed was due to modification of the DIC or ALK gradient within the Miocene ocean (i.e., constant DIC or ALK), then the Miocene surface water pH vs. B(OH)_4_^−^/HCO_3_^−^ would display a relationship with a relatively shallow slope (red and blue lines in Fig. 3). Similarly, if a change in these variables was due to whole-scale change in ALK:DIC with a 2:1 ratio due to carbonate compensation in response to changing the water column gradients, a shallow slope would result (green line in Fig. 3). DIC removal or addition due to the long-term geological processes (e.g., CO_2_ invasion, chemical weathering, and/or C_org_ burial/oxidation) where carbonate compensation operates to maintain a stable CaCO_3_ saturation state can be approximated by the purple line on Fig. 3 where ALK and DIC change with a 1:1 ratio. This results from the addition/removal of 1 mol of CO_2_ and the full carbonate compensation response that adds/removes ALK and DIC with a 2:1 ratio to maintain a steady state of CaCO_3_ burial^37^. The Miocene B(OH)_4_^−^/DIC and pH data show a relationship which closely follows the 1:1 (ALK:DIC) line, suggesting the changes in carbonate system we reconstruct (i.e. pH and B(OH)_4_^−^/HCO_3_^−^) were mainly due to the addition/removal of DIC through slow geological processes, coupled with the expected CaCO_3_ system response (Fig. 3).

This approach allows a semi-quantification of ocean DIC through the Miocene. However, given the inherent uncertainties involved, particularly in the [B]_sw_ and the sensitivity of the B/Ca-[B(OH)_4_^−^/DIC]_sw,_ only relative changes in Miocene DIC are considered here (Fig. 4). Using this semi-quantitative approach, we are able to show that the low B/Ca and B(OH)_4_^−^/HCO_3_^−^ values of the MCO suggest higher DIC levels in this warm interval, and that DIC had declined by ~200 to 400 μmol/kg by the end of the MMCT glaciation (see Methods and Fig. 4). Superimposed on this overall change in DIC on millions of years timescales are higher resolution changes in DIC that were likely orbitally paced. For example, paired δ^11^B and B/Ca records from Malta provide strong evidence for a DIC decline across the CM6 event (Supplementary Fig. 3). Although the absolute values remain poorly constrained we estimate this decline to be on the order of ~300 μmol/kg.

## Discussion

Our planktic foraminiferal δ^13^C_p_ records show the slow rise and peak during the MCO, directly followed by a recovery during the MMCT, typical of the globally recognized MCIE. Given the original Monterey Hypothesis, one would expect the broad increase in δ^13^C associated with the onset of the MCIE to be associated with a decrease in seawater DIC. This is in contrast to our boron-based DIC reconstructions, which show a broad low in B/Ca values, low pH, and hence high DIC throughout the duration of the MCO (Figs. 3 and 4, and see Methods), whereas Mg/Ca-SSTs estimates indicate a ~1.8 °C warming (Fig. 1). These findings are therefore not consistent with an interpretation of increasing organic carbon burial in a cooling climate at the onset of the MCIE. Instead, it is consistent with a volcanic driver initiating the warmth of the MCO, which pushed the climate system into a less glaciated state with higher sea level. The majority of volcanic emissions from the CRB erupted between 16.7 and 15.9 Ma, which is approximately coincident with the onset of the MCO^31^ (Fig. 4). Several studies propose that CRB volcanism supplied the necessary carbon emissions to trigger and sustain higher than modern atmospheric CO_2_ concentrations (~470–630 ppm), and warm MCO climate sufficient to inhibit reglaciation of Antarctica^24,25,29–39^ (Fig. 4).

A rise of atmospheric CO_2_ and positive δ^13^C excursion (MCIE), despite the release of presumably isotopically depleted volcanic carbon (mantle value of −5‰) may at face value seem implausible. However, the higher CO_2_ levels could act to increase ocean δ^13^C by changing the fractionation factor associated with photosynthetic fixation of carbon, offsetting the depleted volcanic CO_2_ contribution^25^. Alternatively, a relatively slow emission rate or a carbon isotope composition heavier than the traditional −5‰ mantle value, possibly through metamorphic degassing of country rock, could more easily reconcile a positive MCIE with increasing CO_2_ levels. Separation of the δ^18^O_b_ signal into temperature and δ^18^O_sw_ shows a decrease in seawater δ^18^O (δ^18^O_sw_)^21^ heading into the MCO suggesting a rise in sea level (Fig. 4). This is supported by numerous records of ice volume and sea-level variability based on sedimentological and oxygen isotope evidence from the early to mid-Miocene, which indicates that the Antarctic ice sheet was highly variable during this interval with equivalent to 30–80% loss of modern Antarctic ice sheet^15,21,30,40–42^. It is therefore conceivable that this glacio-eustatic expansion of shelf area would lead to an enhanced capacity for continental organic carbon burial. As modern continental shelves produce three times the mean primary productivity of the ocean^43^ this can lead to organic-rich low-oxygen sediment conditions ideal for increased preservation of organic carbon during the MCO. Furthermore, shelf facies are typically associated with relatively high sedimentation rates, which also favors organic matter preservation and burial^44^. Despite this proposed propensity for enhanced organic carbon burial, elevated surface ocean DIC levels over millions of years could be sustained by the supply of carbon emissions via Columbia River Basalt eruptions. This mechanism would not be expected to cause an increase in productivity or organic carbon burial in open ocean sites, consistent with evidence from deep-water palaeoproductivity proxies^45^. The MCO sea level rise likely also stimulated enhanced shallow water carbonate deposition in suitable locations, representing an additional source of CO_2_ to the atmosphere^46^. The net effect on the carbon cycle is difficult to quantify as it would depend on the balance between the volcanic and chemical weathering fluxes, and the proportion of organic carbon to carbonate burial fluxes. Nevertheless, our records provide the first evidence to suggest that the MCIE was associated with high oceanic DIC that sustained enhanced organic carbon burial over millions of years (Fig. 4f). Overall, this implies a role for CO_2_ induced glacio-eustatic sea level rise in modulating the organic carbon subcycle and producing the largest carbon isotope excursion of the Neogene. The associated burial of marine organic carbon therefore represents a negative feedback, mitigating further atmospheric CO_2_ rise. We note that prior studies suggest that marine organic carbon accumulation rates within the Monterey Formation (eastern Pacific) are too low to account for the entire magnitude of the MCIE and additional light carbon enriched depocenters were proposed to reconcile the full magnitude of the carbon isotope excursion^45–47^. Here we look at this question with an ocean-based lens and note that additional studies are needed to explore the relative contribution of shelf vs. continental organic carbon sequestration (e.g., enhanced peat formation) in driving the MCIE.

Overall, our results suggest that during the MCO there is an increase in organic carbon burial as seen for the Early Eocene Climatic Optimum^48^ and Cretaceous OAEs^33,34,49^. This played a role in mitigating the volcanically driven rise in CO_2_, acting as a negative feedback. However, in contrast to the MCIE, the hallmark of OAE events are widespread anoxic and euxinic conditions. The greenhouse state during the Cretaceous had virtually no continental ice and widespread warmth^50,51^. Furthermore, more sluggish ocean circulation due to the lack of polar ice and lower ocean oxygen content due to overall warmer ocean temperatures may have led to an increased capacity to produce an OAE in the early Cenozoic/Late Cretaceous. We conclude therefore that the MCIE was likely a failed OAE due to the presence of an Antarctic ice sheet and relatively well-ventilated ocean in an icehouse time interval.

Superimposed on the long-term broad MCIE and its recovery are short-lived (~400 kyr) carbon maxima events (CM1–6) associated with positive excursions in δ^18^O_b_, glaciation/cooling and the long-term component of eccentricity (~400 kyr), with CM6 having the largest amplitude^16–18,20,52^. δ^18^O_b_ leads benthic δ^13^C_b_ in the eccentricity band in Miocene isotopic records indicating a lag of carbon cycle over climate variability^19^. Our planktic B/Ca record at ODP Site 761 exhibits similar short-term variability as δ^18^O_b_ and δ^13^C_b_ that is superimposed on the broad decrease in B/Ca, although the resolution of the record limits our ability to examine its possible orbital signature (Figs. 1, 2, and 4). An increase in B/Ca associated with CM-6 evident from ODP Site 761 and Malta indicates a DIC decline (Supplementary Fig. 3). This suggests that although the broad positive MCIE is associated with relatively high DIC, the individual carbon maxima events are associated with higher B/Ca (lower DIC), implying that different processes are responsible for the two features of the carbon isotope record.

Previous work has shown that CM-6 is associated with an invigoration in ocean circulation and increase in marine export productivity^45,53,54^ and *p*CO_2_ draw-down^23^, following a major period of Antarctic ice sheet expansion^55^. This is a similar situation to the glaciation and positive CIE which occurs at the Oligocene–Miocene boundary (~23 Ma)^56^. As with the MCO, these δ^13^C carbon isotope excursions are associated with enhanced carbon burial and potentially enhanced marine productivity.

Modeling studies have linked the origin of the long-period eccentricity pattern in the δ^13^C record to insolation driven changes in a marine biosphere productivity^57^ and increased weathering and nutrient input and marine productivity^58^. Data-model comparisons show that the magnitude of δ^13^C change and the timing and frequency of CM events depends on a balance between orbitally driven changes in ice volume and hence sea-level and shelf deposition/weathering and marine productivity^53^. Overall, this suggests that CM events during the MCIE were linked to high nutrient delivery sustained by high CO_2_-driven weathering alongside insolation-driven marine productivity events. However, further work is needed to fully consider the global nutrient budget and role of insolation in setting the stage for Miocene CM events.

Our records therefore indicate that increased marine organic carbon burial exhibited contrasting time-scale dependent behavior during the middle Miocene: on short-term orbital timescales it acted as positive climate feedback during the orbitally paced CM cooling events, and on million year timescales it acted as a negative climate feedback, mitigating volcanic CO_2_ emissions during the MCO.

Overall, our records suggest planktic B/Ca is a powerful proxy to explore the oceanic carbon cycle during large carbon cycle perturbation events. Our new DIC record represents the dynamic interaction between volcanic driven CO_2_ input, organic carbon burial, and weathering. The broad δ^13^C increase of the MCIE was associated with high surface ocean DIC concentration and the sustained warmth of the MCO. We propose that elevated carbon emissions associated with Columbia River Basalt Flood volcanism induced global warming and sea level rise, which enhanced organic carbon burial via the drowning of continental shelves. We suggest that the broad MCIE itself is therefore a consequence of this key negative feedback process. The icehouse climate state of the middle Miocene, with its continuous ocean ventilation likely prevented the regime of enhanced organic carbon preservation from developing into a full-blown OAE as seen in the greenhouse world of the Cretaceous. During the CM events of the Miocene, climatic cooling episodes triggered corresponding periods of increased marine productivity, which likely reduced surface ocean DIC and atmospheric CO_2_ and acted as a positive carbon cycle feedback process consistent with the original Monterey Hypothesis. The nature and expression of carbon cycle feedbacks in response to C-addition are therefore state-dependent, and such feedbacks may be critical when exploring future carbon emission scenarios in Earth System models.

## Methods

### Age model and study sites

Samples from DSDP Site 588 and ODP sites 761 Hole B, 926 Holes A/B, and 872 Hole C were used to reconstruct SST and $\left[ {{\mathrm{B(OH)}}_4^ - /{\mathrm{DIC}}} \right]_{{\mathrm{sw}}}$ across the early to middle Miocene (22.0–11.5 Ma; Supplementary Fig. 1). Planktic foraminifera Mg/Ca, B/Ca, and δ^13^C_p_ records from ODP site 761B were sampled at an average temporal resolution of 23 kyr. In contrast, our other sites were sampled at lower resolution for B/Ca: ODP 926 (~200 kyr) and DSDP 588 (~100 kyr) and ODP 872 (~1 Myr). ODP 761 (16°44.23’S, 115°32.10’E, water depth of 2179 m) is situated in the Indian Ocean on the Wombat Plateau. Site ODP 926 (3°43.148’N, 42°54.507’E, water depth 3598 m) in the eastern equatorial Atlantic Ocean on Ceara Rise. Site DSDP 588 A (26°06.7’ S, 161°13.6’ E, water depth 1548 m) in the Southwest Pacific Ocean on Lord Howe Rise. ODP Site 872 (10°N, 162°E, 1287 m water depth) is in the tropical north Pacific gyre on the sedimentary caps of flat-topped seamounts.

The age model for Site 761B is based on a fourth-order polynomial fit through the biostratigraphic and isotopic datums^14,35^. For ODP site 926, we use a polynomial fit through nannofossil and planktic foraminifer biostratigraphic datums adapted to the CK95 timescale^35^. The published age model for DSDP 588^59^ is based on compiled biostratigraphy, magnetostratigraphy, isotope stratigraphy calibrated to CK95. Ages for ODP Site 872 were calculated by linear interpolation between reliable biostratigraphic datums^60^ from the early to mid-Miocene adapted to the CK95 timescale^35^. Core sites^29,30,35,61^ used in this study were selected because currently they are located in regions where modern surface waters are close to equilibrium with respect to atmospheric CO_2_ (Supplementary Fig. 1). We use δ^11^B-pH estimates^35^ from ODP sites 761, 872, and 926 in addition to B/Ca presented in this study to estimate surface ocean $\left[ {{\mathrm{B(OH)}}_4^ - /{\mathrm{DIC}}} \right]_{{\mathrm{sw}}}$ and$\left[ {{\mathrm{B(OH)}}_4^ - /{\mathrm{HCO}}_3 - } \right]_{{\mathrm{sw}}}$.

### Planktic foraminiferal taxonomy and ecology

We generated trace element and isotope records using *T. trilobus*, a multi-chambered, photosymbiont bearing species, which is predominantly a mixed layer dweller calcifying at 0–50 m and is abundant in subtropical and tropical oceans. *T. trilobus* is a morphospecies of *Trilobatus sacculifer*^62,63^ present throughout the Neogene and Quaternary periods. *T. trilobus* has been used extensively in previous Miocene climate and boron isotope studies^29,23,30,35^ due to its narrow habitat range, well-defined δ^11^B-pH calibration, calcification close to equilibrium conditions (i.e., minimal vital effects), and abundance^29,35,63,64^. Further, previous studies demonstrated that temperatures derived from *T. sacculifer* are most suitable for estimating annual mean SST^65^ in tropical waters (between 20° N/S) within ±1 °C. Visual inspection of preservation (Supplementary Fig. 4) of *T. trilobus* from ODP site 761B show moderately good preservation with no visible signs of infilling or dissolution. Visual inspection of foraminiferal specimens from ODP site 926 shows evidence of a recrystallized nature, however the Mg/Ca and boron isotope signature has remained intact^29,35,66^. Visual inspection of foraminiferal specimens from ODP site 872 show a glassy nature indicative of well-preserved specimens and exceptionally good carbonate preservation supported by previous work using scanning electron microscopic (SEM) images^60,67^. Visual inspection of foraminiferal specimens from DSDP 588 have been shown to be pristine in nature with no evidence of dissolution and have been previously used in oxygen isotope reconstructions^52^.

### Trace metal and isotopic analysis

Between 30–40 tests of the planktic foraminifera *T. trilobus* were picked from the 300–355 μm size fraction from ODP site 761, 926, and DSDP 588. ODP 872 samples were prepared for boron isotope analysis and an aliquot of the foraminiferal sample was used to collect trace element data (see ref. ^35^). Picked specimens were weighed, gently crushed between glass plates and homogenized for chemical cleaning and geochemical analysis. In samples where *T. trilobus* abundance was low (~30 samples), fewer specimens (10–20 individuals) were analyzed. After sample homogenization, an aliquot of the foraminiferal sample was used to collect trace element and δ^13^C_p_ data. It is noteworthy that only δ^13^C_p_ from ODP site 761 are presented here.

Test fragments for Mg/Ca and B/Ca analyses were cleaned using a protocol to remove surficial clays, adhered organic matter, and potential secondary carbonate overgrowths^68^. Clay removal consists of repeated rinses with low boron Milli-Q water and methanol. The oxidative cleaning step consists of a sodium hydroxide and hydrogen peroxide solution to remove any present organic matter. The reductive cleaning step was not included in the cleaning process as this step is unnecessary for B/Ca as the cleaning does not alter the B/Ca ratio of foraminifera or calcite bound boron^69^. Lastly, a weak acid leach using 0.001 M hydrochloric acid was conducted to remove any ions re-absorbed during the cleaning process on the test surface. Following the clay removal and oxidative steps, samples were examined under a binocular microscope and visible non-carbonate particles were removed using a fine paintbrush. Cleaned, treated samples were dissolved in trace metal pure 0.065 M HNO_3_ and diluted with trace metal pure 0.5 M HNO_3_ to a final volume of 350 μl, to achieve a target calcium concentration of 4 mM. Trace element analysis (B, Mg, Al, Ca, Mn, Fe) for ODP 761, ODP 926, and DSDP 588 was carried out at Cardiff University on a Thermo Scientific Element XR Sector Field Inductively Coupled Plasma Mass Spectrometer (SF-ICP-MS), whereas samples from ODP site 872 were analyzed at the University of Southampton, using a Thermo Scientific Element 2 XR SF-ICP-MS. In both instances, calcium concentrations of bracketing standards were matched to foraminifera samples to reduce matrix effects^70,71^.

To monitor the possibility of clay contamination Mg/Ca and B/Ca data were rejected if Al/Ca exceeded 80 μmol/mol for all sites. Additional cleaning effectiveness was supported by observing no significant correlation between Mg/Ca, and Fe/Ca and Mn/Ca (Supplementary Figs. 5-6). There is no correlation between B/Ca and Fe/Ca at any of the investigated sites. There is however a positive correlation between Mn/Ca and B/Ca at ODP site 926 (*R*^2^ = 0.4) and 761 (*R*^2^ = 0.6), and a slight negative correlation (*R*^2^ = 0.3) between Mn/Ca and B/Ca at ODP site 872 (Supplementary Fig. 6). However, at site 761, correlations across 500 kyr windows from 16.5 to 11.5 Ma show no significant correlation (*R*^2^ < 0.3; *p* > 0.05) between these variables in 8 out of the 10 intervals (Supplementary Table 1). We therefore propose here that the Mn/Ca record reflects another aspect of the system (e.g., oxygenation, productivity), which would be correlated on geological timescales with B/Ca as in the Miocene^72^. Long-term precision at Cardiff University and University of Southampton was determined by analyzing an independent consistency standard during each run for 1 year. Reported values are 0.5% and 4% (r.s.d.) for Mg/Ca and B/Ca, respectively, for analyses at Cardiff University and 2% and 4% (r.s.d.) for Mg/Ca and B/Ca, respectively, for analyses at University of Southampton.

Stable carbon isotope ratios were measured at Cardiff University on a Finnigan MAT 252 micro-mass spectrometer Kiel III Carbonate Device when sample weights were <100 μg and measured on a Delta isotope ratio mass spectrometer when samples were greater than 100 ug. Long-term precision based on replicate measurements of a laboratory standard (NBS 19) are 0.08‰ for δ^13^C.

### Mg/Ca-paleotemperature calculation and constraining Mg/Ca seawater changes

Planktic *T. trilobus* is the morphotype of the modern *T. sacculifer*, we consider it appropriate to calculate mean annual SSTs from *T. trilobus* Mg/Ca data using the *T. sacculifer* calibration^65^:

1 $$\frac{{Mg}}{{Ca}} = 0.347\ \left( {\!\pm \! 0.011} \right)e^{0.090\left( {\! \pm \! 0.003} \right)T}$$

Variations in Miocene foraminiferal Mg/Ca are driven by changes in temperature; however, on longer timescales, both changes in temperature and in seawater Mg/Ca must be considered. Recently, it has been shown that a power function^73^ best describes this relationship.

2 $$\frac{{Mg}}{{Ca}}_{foram} = \left[ {\frac{{\frac{{Mg}}{{Ca}}_{sw}\left( t \right)}}{{\frac{{Mg}}{{Ca}}_{sw}\left( 0 \right)}}} \right]^CBe^{AT}$$

where Mg/Ca_sw_(t) and Mg/Ca_sw_ (0) are seawater Mg/Ca ratios for the Miocene and present, and A, B, and C are constants (A = exponential, B = pre-exponential, C = power constant), respectively.

Mg and Ca have relatively long residence times (~13 Myr and ~1.1 Myr, respectively) in the ocean. The modern day seawater Mg/Ca value is 5.2 mmol/mol and low-resolution fluid inclusions data^74^ show a rise across the Neogene to the modern values. Here we use the fluid inclusion value of 3.43 mmol/mol in the paleotemperature calculation. We use the *T. sacculifer* calibration^65^ (*A* = 0.09, *B* = 0.347) for *T. trilobus* and apply a power constant^75^ of 0.41 for *T. Sacculifer* based on the available data for this species^76^. *T. trilobus* SST estimates can be calculated from the following equation:

3 $$\frac{{Mg}}{{Ca}}_{foram} = 0.293\,e^{0.090T}$$

Using a range of Mg/Ca seawater estimates, calculated SST during the MCO period in Site 761 ranges from 1.4 °C warmer to uncorrected Mg/Ca-SST estimates being cooler by 0.5 °C than the modern (modern mean annual temperature is ~27.7 °C).

In addition to changes in seawater Mg/Ca, Mg incorporation in foraminifera has shown a species-specific dependency to changes in carbonate chemistry. A recent review^77^ proposed a pH correction was necessary to correct Mg/Ca records when estimating SST for some species. This study showed that Mg/Ca ratios in *T. sacculifer* are insensitive to changes in pH. As Mg/Ca ratios are derived from *T. trilobus* shells, the morphotype of *T. sacculifer*, we apply no pH correction in this study.

### Foraminiferal preservation

Several lines of evidence suggest that dissolution does not significantly affect the Mg/Ca or B/Ca values at Site 761 and suggest our data represent a climate signal. First, ODP Site 761 is situated well above the modern lysocline, above the critical 20 μmol/kg ΔCO_3_^2−^, in a relatively shallow burial depth during the middle Miocene (<50 m) (GLODAP^78^). Average shell weight of *T. trilobus*, from the 300–355 μm size fraction, does not covary with the Mg/Ca or B/Ca record, supporting our argument that these values are not biased (Supplementary Fig. 7).

Despite the reasonable appearance of foraminifera, all tests appear frosty or opaque in contrast to exceptionally well-preserved translucent test shells from hemipelagic muds^79^. However, this preservation state is typical of most deep-sea carbonates and is caused by micro-recrystallization of calcite. Large-scale recrystallization is not evident in SEM images for ODP site 761 (Supplementary Fig. 4), suggesting that diagenesis did not drive prominent shifts in δ^13^C and trace elements. In addition, Sr/Ca ratios from ODP site 761 show high values (~1.1–1.2 mmol/mol; Supplementary Table 2), which are consistent across much of the record, suggesting that diagenesis did not have a major influence on Mg/Ca or B/Ca variations. Nonetheless, Mg/Ca values decrease by a negligible amount with initial diagenetic alteration^79^, thus temporal changes in Mg/Ca are less likely to be affected. Further work^80^ has shown a decrease in planktic B/Ca in recrystallized relative to well-preserved foraminifera; however, additional work is needed to isolate the primary diagenetic signal. For the reasons outlined above, we believe that diagenesis had a minimal effect on our records and we therefore interpret the geochemical records in terms of paleoceanographic conditions.

### Estimation of surface ocean [B(OH)_4_^−^/HCO_3_^−^]_sw_ and [B(OH)_4_^-^/DIC]_sw_ from B/Ca

Similar to boron isotopes, the boron content (expressed as B/Ca) of planktic foraminifera has been suggested to be a proxy for ocean carbonate chemistry^69,81^. Boron exists in seawater primarily as two species borate (B(OH)_4_^−^ and boric acid (B(OH)_3_), and the relative concentration of each boron species and their δ^11^B composition varies with pH in seawater. Boron isotopic evidence and other arguments suggest that B(OH)_4_^−^ is the species predominantly incorporated into the foraminiferal calcite lattice^82^. B/Ca in planktic foraminifera is controlled by a combination of ocean carbonate chemistry parameters (pH, B(OH)_4_^−^ ${\mathrm{HCO}}_3^ -$ and DIC) as demonstrated through empirical culture experiments and field studies^71,81,83^. Culturing efforts aimed to disentangle this co-varying carbonate system parameters show that B/Ca is governed principally by the ratio of seawater B(OH)_4_^−^ to DIC or ${\mathrm{HCO}}_3^ -$^81,84^. Application of the B/Ca proxy to reconstruct shifts in the concentration of [B(OH)_4_^−^/DIC] in seawater across major climate transitions has shed light on past carbon cycle perturbations^84,85^ (e.g., Paleoceane-Eocene Thermal Maximum), where large shifts in the ocean carbonate system occurred. Similarly, in our study we aim to examine the carbon cycle perturbations of the early to middle Miocene, a time interval whereby a large carbonate system shifts likely occurred, suggesting some of the complexities that others have discussed with regard to the glacial–interglacial cycles may not be significant.

Culture experiments with living *T. sacculifer* demonstrate a positive relationship between B/Ca and oceanic carbonate system parameters, where B/Ca increases at higher pH and lower DIC and HCO_3_^−^ concentrations (Supplementary Fig. 8)^81^. These experiments also provide calibrations that relate B/Ca to both [B(OH)_4_^−^/HCO_3_^−^]_sw_ and [B(OH)_4_^−^/DIC]_sw_; however, they argue for a better fit to the latter parameter. Recent culture work further explored the mechanistic understanding of B uptake, indicating that planktic B/Ca, *Orbulina universa* specifically, is driven by HCO_3_^−^ in support of [B(OH)_4_^−^/HCO_3_^−^]_sw_ rather than [B(OH)_4_^−^/DIC]_sw_ controlling B/Ca^83^. Here we present [B(OH)_4_^−^/HCO_3_^–^]_sw_ to evaluate the main drivers of carbonate system change through the MCIE. Further, we consider both B/Ca carbonate chemistry sensitivities (e.g., [B(OH)_4_^−^/HCO_3_^−^]_sw_ and [B(OH)_4_^−^/DIC]_sw_) to estimate surface ocean DIC.

When we compile all available B/Ca-[B(OH)_4_^−^/DIC]_sw_ and [B(OH)_4_^−^/HCO_3_^−^]_sw_ *T. sacculifer* calibration data, an offset is observed between culture^81^ and core-top^86^ datasets, where the core-top data sits lower and with a slightly steeper slope predicted by culture experiments, although this slope difference may largely be a function of the different ranges spanned by the datasets, as the available core-top data are limited by the range in surface ocean carbonate chemistry (e.g., [B(OH)_4_^−^/DIC]_sw_) that span the depth habitat of wild-type *T. sacculifer* compared with conditions manipulated in culture work^81^. We account for differences in test size^87^ and normalize values to a common salinity (*S* = 35)^81^, but an offset and slope difference between the datasets remain (Supplementary Fig. 8). Here we suggest the offset could be related to the fact core-top foraminiferal specimens, during their life cycle, sink to water with lower pH and higher DIC/ HCO_3_^−^ in contrast to the cultured specimens. This is supported by the lower core-top B/Ca values relative to culture. Furthermore, the core-top study used *T. sacculifer* specimens with the final sac-life chamber, which have lower B/Ca relative to non-sac chambers^81^, in contrast to the cultured specimens.

To address the B/Ca offset between datasets we retain the slope predicted in culture and normalize the culture to core-top values to generate new ad hoc B/Ca to [B(OH)_4_^−^/DIC]_sw_ and [B(OH)_4_^−^/HCO_3_^−^]_sw_ calibrations (B/Ca = 33 + 773*[B(OH)_4_^−^/DIC]_sw_;B/Ca = 39 + 561*[B(OH)_4_^−^/HCO_3_^−^]_sw_; Supplementary Fig. 8). Application of these ad hoc calibrations to ODP 761 B/Ca reconstructions requires additional consideration of past changes in seawater boron ([B_sw_]) on calibration sensitivity. We can assume a constant seawater composition over the studied interval as it is considerably shorter than the oceanic residence time of B (10–20 million years)^88^. A positive relationship between seawater B concentration ([B_sw_]) and B content in planktic foraminiferal calcite (*O. universa*) is observed in culture experiments^81,83,84^ (Supplementary Fig. 9). Following Mg/Ca convention and the observation that Mg/Ca sensitivity changes as a function of seawater Mg/Ca ratio, we adjust the calibration sensitivity to changes in B/Ca_sw_, to account for the influence of variable Mg/Ca_sw_ on Mg/Ca thermometry^70,75^ by assuming a linear scaling:

4 $$\frac{B}{{C{\mathrm{{a}}}}}{\mathrm{{foram}}} = \frac{{\frac{B}{{C{\mathrm{{a}}}}}sw\,t = t}}{{\frac{B}{{C{\mathrm{{a}}}}}sw\,t = 0}}x\frac{{B({\mathrm{{OH}}})_4^ - }}{{{\mathrm{{HCO}}}_3^ - }} \ast {m} + {b}$$

5 $$\frac{B}{{C{\mathrm{{a}}}}}{\mathrm{{foram}}} = \frac{{\frac{B}{{C{\mathrm{{a}}}}}sw\,t = t}}{{\frac{B}{{C{\mathrm{{a}}}}}sw\,t = 0}}x\frac{{B({\mathrm{{OH}}})_4^ - }}{{{\mathrm{{DIC}}}}} \ast {m} + {b}$$

where *B/Ca*_*sw*_ *(t* *=* *t)* for the age of the sample and *B/Ca*_*sw*_ *(t* *=* *0)* is modern B/Ca_sw_ and *m* and *b* are the slope and y-intercept for each calibration from. Miocene estimates of B_sw_ and Ca_sw_ are derived from boron isotope modeling and fluid inclusion estimates, respectively^74,88^.

Calculation of DIC and HCO_3_^−^from the B/Ca-[B(OH)_4_^−^/DIC]_sw_ and [B(OH)_4_^−^/HCO_3_^−^]_sw_ calibrations requires an independent estimate of B(OH)_4_^−^ across the Miocene, and here we use δ^11^B-pH estimates from ODP 761, 872, and 926^35^ to calculate B(OH)_4_^−^. B(OH)_4_^−^ is related to pH as described here:

6 $${\mathrm{{pH}}} = - \log [H^ + ]$$

7 $$B({\mathrm{{OH}}})_4^ - = B_{{\mathrm{{TOT}}}}/\left( {1 + \frac{{[H^ + ]}}{{K_{\mathrm{{B}}}^ \ast }}} \right)$$

where $K_{\mathrm{{B}}}^ \ast$ is the stoichiometric equilibrium constant for boric acid^89^ at time equivalent *T*, *S*, and *P*, and Mg/Ca_sw_ (*t* = *X* Ma). pH estimates used were derived from the three δ^11^B_sw_ scenarios^35^ (G17, RH13, L02). The pH derived from each scenario have a similar structure but differ in absolute pH values. This should minimally impact the DIC trend across the Miocene, however, we use pH estimates from all scenarios to estimate the likely DIC (Supplementary Fig. 10). Surface ocean pH estimates from ODP 872 are higher by 0.1 pH units compared with site 926 and 761 in the early Miocene (16.5–17.0 Ma) (Supplementary Fig. 2). To account for this offset, we adjust ODP site 872 by 0.1 pH units and use these adjusted pH values in the DIC estimates. SST estimates are derived from Mg/Ca in complementary samples^35^ and modern salinity is assumed for the entire duration of the Miocene record. We normalize planktic B/Ca values to a common salinity, assuming modern salinity values for each site (*S* = 35)^81^. B_sw_ varied in time according to marine boron isotope budget^88^ in the same manner as above for B/Ca_sw_ estimates. Using the B/Ca-[B(OH)_4_^−^/HCO_3_^−^]_sw_ calibration, we estimate DIC from HCO_3_^−^ using the following equation:

8 $${\mathrm{{DIC}}} = \frac{{[{\mathrm{{H}}}^ + ][{\mathrm{{HCO}}}_3^ - ]}}{{K_1}} + \left[ {{\mathrm{{HCO}}}_3^ - } \right] + \frac{{K_2[{\mathrm{{HCO}}}_3^ - ]}}{{[{\mathrm{{H}}}^ + ]}}$$

where *K*_1_ and *K*_2_ are the carbonic acid dissociation constants^89^.

The principal uncertainties in calculating [DIC]_sw_ and [HCO_3_^−^] in this way were judged to be uncertainties in the B/Ca calibration and in the pH used to calculate [B(OH)_4_^−^]. Here we used Monte Carlo method to propagate these uncertainties into our final estimates of DIC and HCO_3_^−^. In order to highlight the long-term trends in DIC and HCO_3_^−^, we then fit smoothing splines through the data with the degree of smoothing determined by generalized cross validation. Again, the uncertainty in these smoothed trends was determined using a Monte Carlo approach and a consideration of the error bars of each individual estimate.

DIC estimates derived using the range of pH scenarios (G17 v RH13 v L02) show a similar trend across the middle Miocene (Supplementary Fig. 11). Miocene estimates of surface ocean DIC derived from ODP site 761 using both B/Ca-[B(OH)_4_^−^/DIC]_sw_ and [B(OH)_4_^−^/HCO_3_^−^]_sw_ calibrations, and pH estimates from all scenarios show higher DIC during the MCO (14.0–17.0 Ma; Supplementary Fig. 11 and Supplementary Tables 3 and 4; 68% CI; *n* = 15). Early Miocene (17.0–22.0 Ma) estimates are lower than the MCO (Supplementary Table 3; 68% CI; *n* = 7) increasing towards the onset of the MCO. MCO DIC estimates decline following the MMCT. MMCT DIC levels (13.5–11.0 Ma) are lower than the MCO (Supplementary Table 3; 68% Confidence Interval; *n* = 12). Further, the overall decline in DIC from the MCO to post-MMCT of ~220 to 370 μmol/kg (B/Ca-[B(OH)_4_^−^/DIC]_sw_) and 280 to 430 μmol/kg (B/Ca-[B(OH)_4_^−^/HCO_3_^−^]_sw)_ (Supplementary Table 5) is similar to change derived from a modeling study^25^, although absolute values are offset. Absolute Miocene DIC estimates here fall within the general estimates based on a range of approaches from modeling to geochemical reconstructions (Supplementary Fig. 12)^35,90,91^.

Regardless of the approach and location, our findings suggest the MCO DIC levels were higher than the early Miocene and late middle Miocene and DIC levels decreased across the MMCT by as much as 200–400 μmol/kg. Given the inherent uncertainties in these calculations, particularly in the [B]_sw_ and the sensitivity of the B/Ca-[B(OH)_4_^−^/DIC]_sw_ and [B(OH)_4_^−^/HCO_3_^−^]_sw_, the absolute values should be taken with caution. The trends recognized, however, because they are largely determined by B/Ca can be considered robust.

Although the Miocene B/Ca record from ODP site 761 presented in this study encompasses the short-term variations in δ^13^C (i.e., CM events), due to the lack of complimentary δ^11^B-pH records at the same resolution, we are unable to estimate relative DIC on these timescales, based on records from this study (Supplementary Fig. 3). Here we look to the *T. trilobus* B/Ca record from Malta which includes both B/Ca and δ^11^B-pH estimates across CM6 and shows a similar increase in B/Ca (Supplementary Fig. 3). Using the same approach as above and equations 4-7, we estimate relative change in DIC across CM6. A 17 μmol/mol increase in planktic B/Ca and corresponding increase of 0.08 in pH (Supplementary Fig. 3) across the CM6 event equates to a lowering of DIC by ~300 μmol/kg, although in absolute terms this remains poorly constrained at present. We do note that to fully quantify and assess surface DIC changes on these timescales, a suite of orbitally resolved records of the carbonate system (i.e., B/Ca and δ^11^B) across mid-Miocene carbon isotope excursions are needed.

## Supplementary information

Supplementary Dataset 1

Supplementary Dataset 2

Supplementary Dataset 3

Supplementary Dataset 4

Supplementary Information

## Data Availability

All data generated during this study supporting its findings are supplied in supplementary data files.

## References

[1] Walker JCG, Hays PB, Kasting JF (1981). A negative feedback mechanism for the long-term stabilization of earths surface-temperature. J. Geophys. Res. Oceans.

[2] Berner RA, Lasaga AC, Garrels RM (1983). The carbonate-silicate geochemical cycle and its effect on atmospheric carbon-dioxide over the past 100 million years. Am. J. Sci..

[3] Raymo ME, Ruddiman WF (1992). Tectonic forcing of late cenozoic climate. Nature.

[4] Chamberlin TC (1899). An attempt to frame a working hypothesis of the cause of glacial periods on an atmospheric basis. J. Geol..

[5] Pagani M, Caldeira K, Berner R, Beerling DJ (2009). The role of terrestrial plants in limiting atmospheric CO2 decline over the past 24 million years. Nature.

[6] Raymo ME (1994). The Himalayas, organic-carbon burial, and climate in the miocene. Paleoceanography.

[7] Derry LA, FranceLanord C (1996). Neogene growth of the sedimentary organic carbon reservoir. Paleoceanography.

[8] Rohling EJ (2012). Making sense of palaeoclimate sensitivity. Nature.

[9] Gibbs SJ (2016). Ocean warming, not acidification, controlled coccolithophore response during past greenhouse climate change. Geology.

[10] Martinez-Garcia A (2014). Iron fertilization of the subantarctic ocean during the Last Ice Age. Science.

[11] Torres MA, Moosdorf N, Hartmann J, Adkins JF, West AJ (2017). Glacial weathering, sulfide oxidation, and global carbon cycle feedbacks. Proc. Natl Acad. Sci. USA.

[12] Hoenisch B (2012). The geological record of ocean acidification. Science.

[13] Heinze C (2018). Climate feedbacks in the Earth system and prospects for their evaluation. Earth Syst. Dynam. Discuss.

[14] Holbourn A, Kuhnt W, Simo JA, Li QY (2004). Middle miocene isotope stratigraphy and paleoceanographic evolution of the northwest and southwest Australian margins (Wombat Plateau and Great Australian Bight). Palaeogeogr. Palaeoclimatol. Palaeoecol..

[15] Levy R (2016). Antarctic ice sheet sensitivity to atmospheric CO_2_ variations in the early to mid-Miocene. Proc. Natl Acad. Sci. USA.

[16] Woodruff F, Savin SM (1985). Delta-C-13 values of miocene pacific benthic foraminifera - correlations with sea-level and biological productivity. Geology.

[17] Holbourn A, Kuhnt W, Schulz M, Flores JA, Andersen N (2007). Orbitally-paced climate evolution during the middle Miocene “Monterey” carbon-isotope excursion. Earth Planet. Sci. Lett..

[18] Savin SM (1985). The evolution of miocene surface and near-surface marine temperatures - oxygen isotopic evidence. Geol. Soc. Am. Mem..

[19] Vincent E, Killingley JS, Berger WH (1985). Miocene oxygen and carbon isotope stratigraphy of the tropical indian-ocean. Geol. Soc. Am. Mem..

[20] Woodruff F, Savin SM (1991). Mid-miocene isotope stratigraphy in the deep sea: high-resolution correlations, paleoclimatic cycles, and sediment preservation. Paleoceanography.

[21] Lear, C. H., Mawbey, E. M. & Rosenthal, Y. Cenozoic benthic foraminiferal Mg/Ca and Li/Ca records: toward unlocking temperatures and saturation states. *Paleoceanography* **25**, 10.1029/2009pa001880 (2010).

[22] Lewis AR, Marchant DR, Ashworth AC, Hemming SR, Machlus ML (2007). Major middle Miocene global climate change: evidence from East Antarctica and the Transantarctic Mountains. Geol. Soc. Am. Bull..

[23] Badger, M. P. S. et al. CO_2_ drawdown following the middle Miocene expansion of the Antarctic Ice Sheet. *Paleoceanography* **28**, 10.1002/palo.20015 (2013).

[24] Hodell DA, Woodruff F (1994). Variations in the strontium isotopic ratio of seawater during the miocene - stratigraphic and geochemical implications. Paleoceanography.

[25] Armstrong McKay DI, Tyrrell T, Wilson PA, Foster GL (2014). Estimating the impact of the cryptic degassing of Large Igneous Provinces: a mid-Miocene case-study. Earth Planet. Sci. Lett..

[26] Pagani M, Arthur MA, Freeman KH (1999). Miocene evolution of atmospheric carbon dioxide. Paleoceanography.

[27] Loutit TS, Pisias NG, Kennett JP (1983). Pacific miocene carbon isotope stratigraphy using benthic foraminifera. Earth Planet. Sci. Lett..

[28] Zhang YG, Pagani M, Liu Z (2014). A 12-million-year temperature history of the tropical Pacific Ocean. Science.

[29] Foster GL, Lear CH, Rae JWB (2012). The evolution of pCO_2_, ice volume and climate during the middle Miocene. Earth Planet. Sci. Lett..

[30] Greenop R, Foster GL, Wilson PA, Lear CH (2014). Middle Miocene climate instability associated with high-amplitude CO_2_ variability. Paleoceanography.

[31] Kasbohm, J. & Schoene, B. Rapid eruption of the Columbia River flood basalt and correlation with the mid-Miocene climate optimum. *Sci. Adv.* **4**, 10.1126/sciadv.aat8223 (2018). 10.1126/sciadv.aat8223 PMC6154988 30255148

[32] Kuerschner WM, Kvacek Z, Dilcher DL (2008). The impact of Miocene atmospheric carbon dioxide fluctuations on climate and the evolution of terrestrial ecosystems. Proc. Natl Acad. Sci. USA.

[33] Jenkyns, H. C. Geochemistry of oceanic anoxic events. *Geochem. Geophys. Geosyst.* **11**, 10.1029/2009gc002788 (2010).

[34] Holbourn A, Kuhnt W, Kochhann KGD, Andersen N, Meier KJS (2015). Global perturbation of the carbon cycle at the onset of the Miocene Climatic Optimum. Geology.

[35] Sosdian SM (2018). Constraining the evolution of Neogene ocean carbonate chemistry using the boron isotope pH proxy. Earth Planet. Sci. Lett..

[36] Abels, H. A. et al. Long-period orbital control on middle Miocene global cooling: integrated stratigraphy and astronomical tuning of the Blue Clay Formation on Malta. *Paleoceanography* **20**, 10.1029/2004pa001129 (2005).

[37] Zeebe, R. & Wolf-Gladrow. *CO*_*2*_*in Seawater: Equilibrium, Kinetics, Isotopes*. (2001).

[38] Super JR (2018). North Atlantic temperature and pCO_2_ coupling in the early-middle Miocene. Geology.

[39] Retallack GJ (2009). Refining a pedogenic-carbonate CO_2_ paleobarometer to quantify a middle Miocene greenhouse spike. Palaeogeogr. Palaeoclimatol. Palaeoecol..

[40] John CM (2011). Timing and magnitude of Miocene eustasy derived from the mixed siliciclastic-carbonate stratigraphic record of the northeastern Australian margin. Earth Planet. Sci. Lett..

[41] Shevenell, A. E., Kennett, J. P. & Lea, D. W. Middle Miocene ice sheet dynamics, deep-sea temperatures, and carbon cycling: a Southern Ocean perspective. *Geochem. Geophys. Geosyst.* **9**, 10.1029/2007gc001736 (2008).

[42] Gasson E, DeConto RM, Pollard D, Levy RH (2016). Dynamic Antarctic ice sheet during the early to mid-Miocene. Proc. Natl Acad. Sci. USA.

[43] Simpson, J. H. & Sharples, J. *An Introduction to the Physical and Biological Oceanography of Shelf Seas* (Cambridge Univ. Press, 2012).

[44] FranceLanord C, Derry LA (1997). Organic carbon burial forcing of the carbon cycle from Himalayan erosion. Nature.

[45] Diester-Haass, L. et al. Mid-Miocene paleoproductivity in the Atlantic Ocean and implications for the global carbon cycle. *Paleoceanography* **24**, 10.1029/2008pa001605 (2009).

[46] Opdyke BN, Walker JCG (1992). Return of the coral-reef hypothesis - basin to shelf partitioning of caco3 and its effect on atmospheric CO_2_. Geology.

[47] Follmi KB (2005). Phosphogenesis and organic-carbon preservation in the miocene monterey formation at Naples beach, California - the monterey hypothesis revisited. Geol. Soc. Am. Bull..

[48] Lauretano V, Zachos JC, Lourens LJ (2018). Orbitally paced carbon and deep-sea temperature changes at the peaK of the early Eocene Climatic Optimum. Paleoceanogr. Paleoclimatol..

[49] Gutjahr M (2017). Very large release of mostly volcanic carbon during the Palaeocene-Eocene Thermal Maximum. Nature.

[50] Miller KG (2005). The phanerozoic record of global sea-level change. Science.

[51] O’Brien CL (2017). Cretaceous sea-surface temperature evolution: constraints from TEX_86_ and planktonic foraminiferal oxygen isotopes. Earth Sci. Rev..

[52] Flower BP, Kennett JP (1993). M iddle miocene ocean-climate transition - high-resolution oxygen and carbon isotopic records from Deep Sea Drilling Project site 588A, southwest pacific. Paleoceanography.

[53] Diester-Haass L (2013). Paleoproductivity during the middle Miocene carbon isotope events: a data-model approach. Paleoceanography.

[54] Holbourn A (2014). Middle Miocene climate cooling linked to intensification of eastern equatorial Pacific upwelling. Geology.

[55] Lear CH (2015). Neogene ice volume and ocean temperatures: Insights from infaunal foraminiferal Mg/Ca paleothermometry. Paleoceanography.

[56] Mawbey EM, Lear CH (2013). Carbon cycle feedbacks during the Oligocene-Miocene transient glaciation. Geology.

[57] Palike H (2006). The heartbeat of the oligocene climate system. Science.

[58] Ma, W. T., Tian, J., Li, Q. Y. & Wang, P. X. Simulation of long eccentricity (400-kyr) cycle in ocean carbon reservoir during Miocene Climate Optimum: weathering and nutrient response to orbital change. *Geophys. Res. Lett.* **38**, 10.1029/2011gl047680 (2011).

[59] Party, S. S. *Site 926* pp. 153–232 (Ocean Drilling Program, College Station, TX, 1995).

[60] Pearson PN (1995). Planktonic foraminifer biostratigraphy and the development of pelagic caps on guyots in the Marshall Islands group. Proc. Ocean Drill. Program Sci. Results.

[61] Tripati AK, Roberts CD, Eagle RA (2009). Coupling of CO_2_ and Ice Sheet Stability Over Major Climate Transitions of the Last 20 Million Years. Science.

[62] Kennett, J. P. & Srinavasan, M. S. Neogene Planktonic Foramifera: a phylogenetic atlas (Hutchinson Ross Publishing Company, Stroudsburg, PA, 1983).

[63] Spezzaferri, S. et al. Fossil and genetic evidence for the polyphyletic nature of the planktonic foraminifera “Globigerinoides”, and description of the new genus *Trilobatus*. *PLoS ONE* **10**, 10.1371/journal.pone.0128108 (2015). 10.1371/journal.pone.0128108 PMC4447400 26020968

[64] Seki O (2010). Alkenone and boron-based Pliocene pCO_2_ records. Earth Planet. Sci. Lett..

[65] Anand, P., Elderfield, H. & Conte, M. H. Calibration of Mg/Ca thermometry in planktonic foraminifera from a sediment trap time series. *Paleoceanography* **18**, 10.1029/2002kpa000846 (2003).

[66] Greenop R (2017). A record of Neogene seawater delta B-11 reconstructed from paired delta B-11 analyses on benthic and planktic foraminifera. Climate.

[67] Pearson PN, Palmer MR (2000). Atmospheric carbon dioxide concentrations over the past 60 million years. Nature.

[68] Barker, S., Greaves, M. & Elderfield, H. A study of cleaning procedures used for foraminiferal Mg/Ca paleothermometry. *Geochem. Geophys. Geosyst.* **4**, 10.1029/2003gc000559 (2003).

[69] Yu, J. M., Elderfield, H. & Honisch, B. B/Ca in planktonic foraminifera as a proxy for surface seawater pH. *Paleoceanography* **22**, 10.1029/2006pa001347 (2007).

[70] Lear CH, Rosenthal Y, Slowey N (2002). Benthic foraminiferal Mg/Ca-paleothermometry: a revised core-top calibration. Geochimica Et. Cosmochimica Acta.

[71] Henehan MJ (2015). Evaluating the utility of B/Ca ratios in planktic foraminifera as a proxy for the carbonate system: a case study of Globigerinoides ruber. Geochem. Geophys. Geosyst..

[72] Klinkhammer, G. P., Mix, A. C. & Haley, B. A. Increased dissolved terrestrial input to the coastal ocean during the last deglaciation. *Geochem. Geophys. Geosyst.* **10**, 10.1029/2008gc002219 (2009).

[73] Hasiuk FJ, Lohmann KC (2010). Application of calcite Mg partitioning functions to the reconstruction of paleocean Mg/Ca. Geochim. Cosmochim. Acta.

[74] Horita J, Zimmermann H, Holland HD (2002). Chemical evolution of seawater during the Phanerozoic: implications from the record of marine evaporites. Geochim. Cosmochim. Acta.

[75] Evans, D. & Mueller, W. Deep time foraminifera Mg/Ca paleothermometry: nonlinear correction for secular change in seawater Mg/Ca. *Paleoceanography* **27**, 10.1029/2012pa002315 (2012).

[76] Delaney ML, Be AWH, Boyle EA (1985). LI, SR, MG, and NA in foraminiferal calcite shells from laboratory culture, sediment traps, and sediment cores. Ge.

[77] Gray WR, Evans D (2019). Nonthermal influences on Mg/Ca in planktonic foraminifera: a review of culture studies and application to the Last Glacial Maximum. Paleoceanogr. Paleoclimatol..

[78] Key, R. M. et al. A global ocean carbon climatology: results from Global Data Analysis Project (GLODAP). *Global Biogeochem. Cycles* **18**, 10.1029/2004gb002247 (2004).

[79] Sexton, P. F., Wilson, P. A. & Pearson, P. N. Microstructural and geochemical perspectives on planktic foraminiferal preservation: “Glassy” versus “Frosty”. *Geochem. Geophys. Geosyst.* **7**, 10.1029/2006gc001291 (2006).

[80] Edgar KM, Anagnostou E, Pearson PN, Foster GL (2015). Assessing the impact of diagenesis on delta B-11, delta C-13, delta O-18, Sr/Ca and B/Ca values in fossil planktic foraminiferal calcite. Geochim. Cosmochim. Acta.

[81] Allen KA, Honisch B, Eggins SM, Rosenthal Y (2012). Environmental controls on B/Ca in calcite tests of the tropical planktic foraminifer species Globigerinoides ruber and Globigerinoides sacculifer. Earth Planet. Sci. Lett..

[82] Hemming NG, Hanson GN (1992). Boron isotopic composition and concentration in modern marine carbonates. Geochim. Cosmochim. Acta.

[83] Howes EL (2017). Decoupled carbonate chemistry controls on the incorporation of boron into *Orbulina universa*. Biogeosciences.

[84] Haynes LL (2017). Calibration of the B/Ca proxy in the planktic foraminifer *Orbulina universa* to Paleocene seawater conditions. Paleoceanography.

[85] Babila, T. L. et al. Capturing the global signature of surface ocean acidification during the Palaeocene–Eocene Thermal Maximum. *Philosophical Transactions of the Royal Society A: Mathematical, Physical and Engineering Sciences* **376.2130**, 20170072 (2018). 10.1098/rsta.2017.0072 PMC6127385 30177558

[86] Foster GL (2008). Seawater pH, _P_CO_2_ and CO_3_^2-^ variations in the Caribbean Sea over the last 130 kyr: A boron isotope and B/Ca study of planktic forminifera. Earth Planet. Sci. Lett..

[87] Ni, Y. Y. et al. A core top assessment of proxies for the ocean carbonate system in surface-dwelling foraminifers. *Paleoceanography* **22**, 10.1029/2006pa001337 (2007).

[88] Lemarchand D, Gaillardet J, Lewin E, Allegre CJ (2002). Boron isotope systematics in large rivers: implications for the marine boron budget and paleo-pH reconstruction over the Cenozoic. Chem. Geol..

[89] Hain MP, Sigman DM, Higgins JA, Haug GH (2015). The effects of secular calcium and magnesium concentration changes on the thermodynamics of seawater acid/base chemistry: Implications for Eocene and Cretaceous ocean carbon chemistry and buffering. Glob. Biogeochem. Cycles.

[90] Ridgwell A (2005). A mid Mesozoic revolution in the regulation of ocean chemistry. Mar. Geol..

[91] Boudreau BP, Middelburg JJ, Sluijs A, van der Ploeg R (2019). Secular variations in the carbonate chemistry of the oceans over the Cenozoic. Earth Planet. Sci. Lett..

[92] Raitzsch M, Honisch B (2013). Cenozoic boron isotope variations in benthic foraminifers. Geology.

[93] Hooper PR, Binger GB, Lees KR (2002). Ages of the Steens and Columbia River flood basalts and their relationship to extension-related calc-alkalic volcanism in eastern Oregon. Geol. Soc. Am. Bull..

[94] Barron, J. A. in *Siliceous Microfossil and Microplankton of the Monterey Formation and Modern Analogs* 105–120 (Pacific Section SEPM, 1986).

[95] Holdgate GR (2007). The middle Miocene Yallourn coal seam - the last coal in Australia the last. Int. J. Coal Geol..

