# Supplementary material for: Ocean Carbon Storage across the middle Miocene: a new interpretation for the Monterey Event
Source: Nat Commun. 2020 Jan 9;11:134. doi: 10.1038/s41467-019-13792-0 (PMC6952451; doi:10.1038/s41467-019-13792-0)
Supplement: Supplementary file 5 — Supplementary Information [file 41467_2019_13792_MOESM5_ESM.pdf]

**Supplementary Information for:**

Ocean Carbon Storage across the middle Miocene: A new interpretation for the Monterey Event

By S.M. Sosdian, T.L. Babila, R. Greenop, G.L. Foster, C.H. Lear

## Supplementary Figures

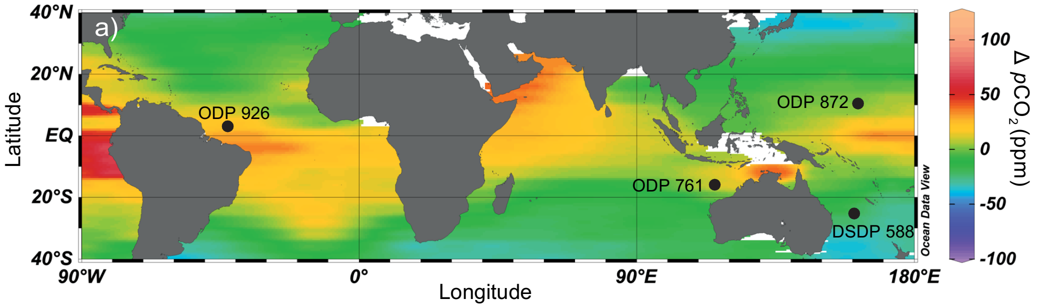


**Supplementary Figure 1** Mean annual air–sea disequilibria with respect to $pCO_2$[1] corrected for anthropogenic DIC invasion[2] showing modern locations of ODP/DSDP sites used in this study (black circles). The modern extent of disequilibria at all sites is <25 ppm $pCO_2$. Surface ocean disequilibria plot made using Ocean Data View.

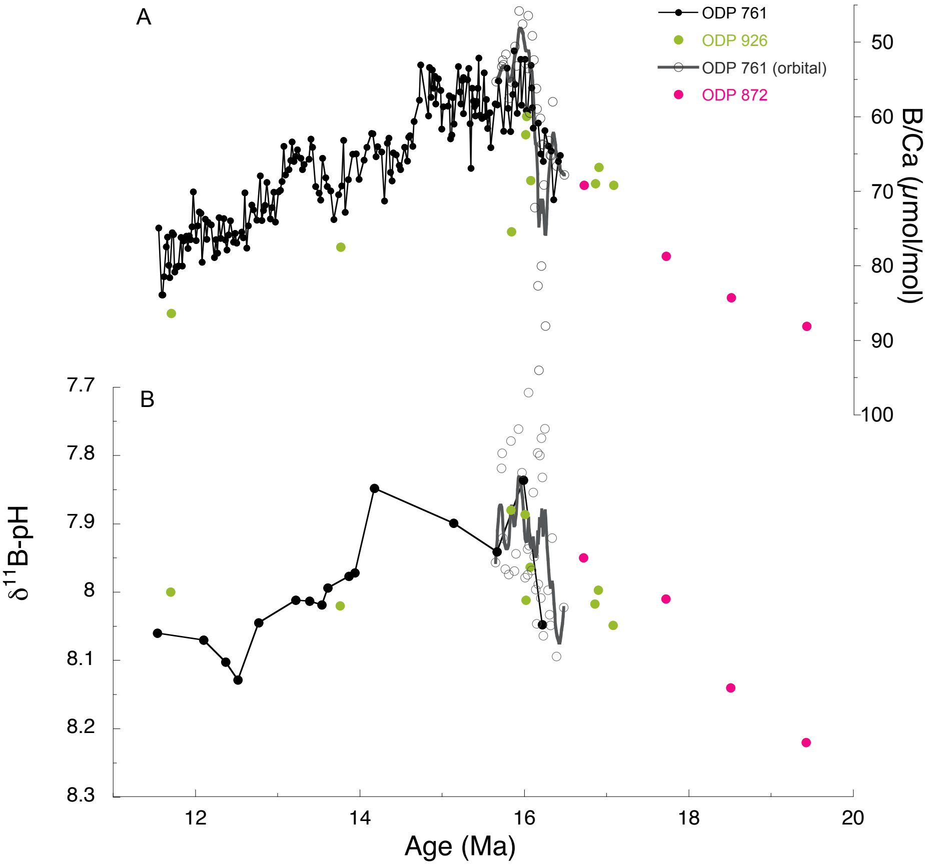


**Supplementary Figure 2** (A) ODP 761, 872, and 926 planktic B/Ca record alongside (B) boron isotope-based surface ocean pH estimates from the same sample. The G17 $\delta^{11}B_{sw}$ scenario[3] was used to estimated DIC using both B/Ca-$[B(OH)_4^-/DIC]_{sw}$ and $[B(OH)_4^-/HCO_3^-]_{sw}$ calibrations.

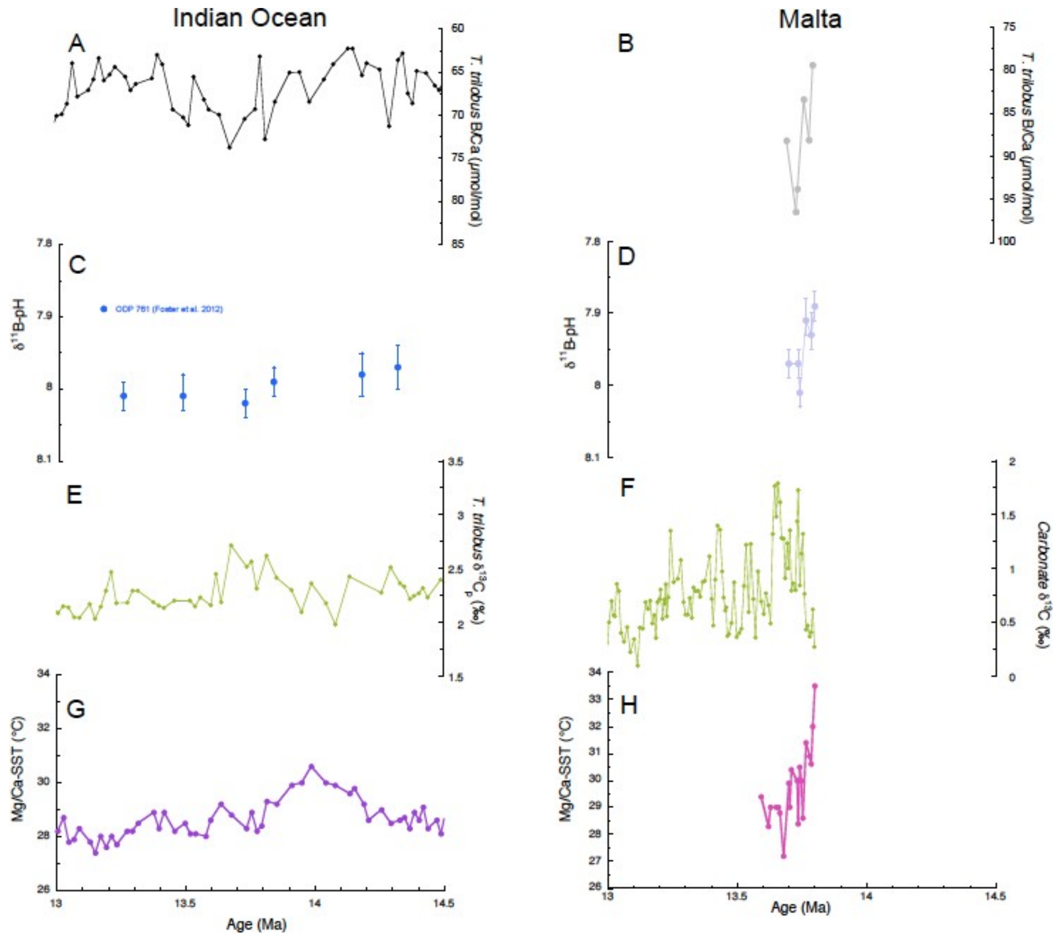


**Supplementary Figure 3** Isotope and trace metal records across CM6 (A, B) *T. trilobus* B/Ca records alongside (C, D) boron isotope based surface ocean pH estimates, and (E, F) carbon isotope records and (G, H) Mg/Ca-derived SST estimates. A,C,E,G are records from ODP 761 and B,D,F,H are published records from Malta[4]. Note the records derived from the Blue Clay Formation of Malta are of orbital resolution across CM6 and on their original published age model[4].

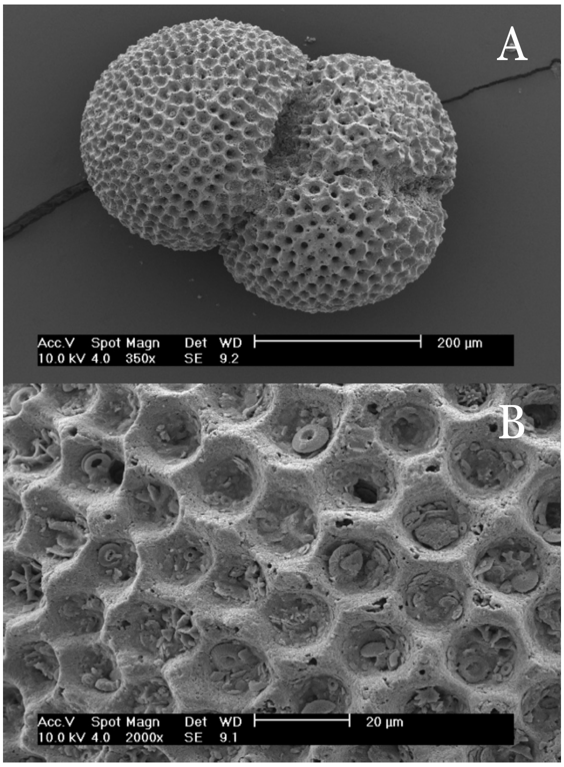


**Supplementary Figure 4** SEM images of species used in this study. (A) Whole test of *Trilobatus trilobus* from ODP 761B 05-05 103-105 cm 300-355 um size fraction (39.73 m below seafloor); (B) Wall structure of test showing original microstructure.

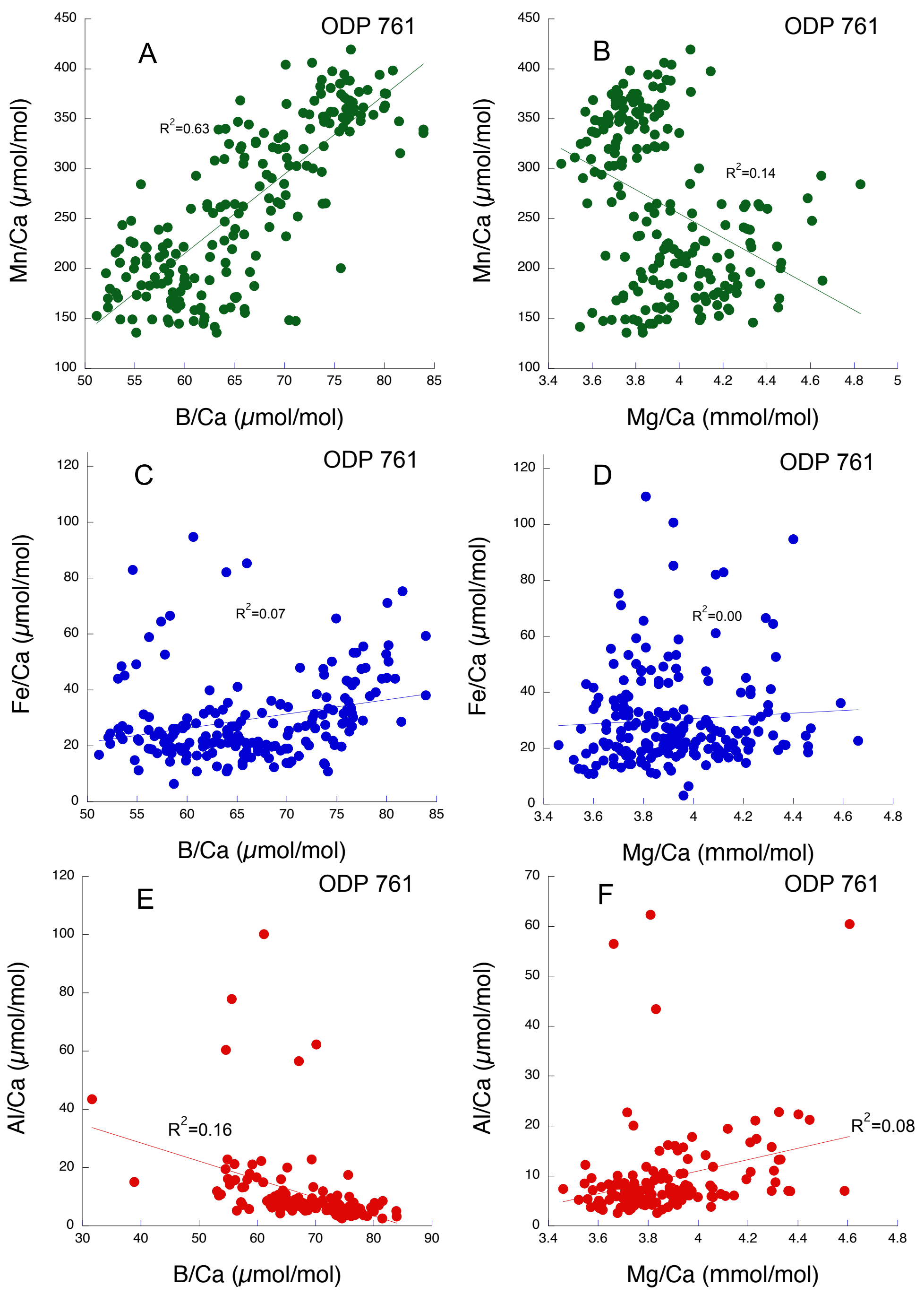


**Supplementary Figure 5** Miocene *T. trilobus* cross-plots of B/Ca and Mg/Ca and (A, B) Mn/Ca, (C, D) Fe/Ca and (E, F) Al/Ca ratios from ODP site 761.

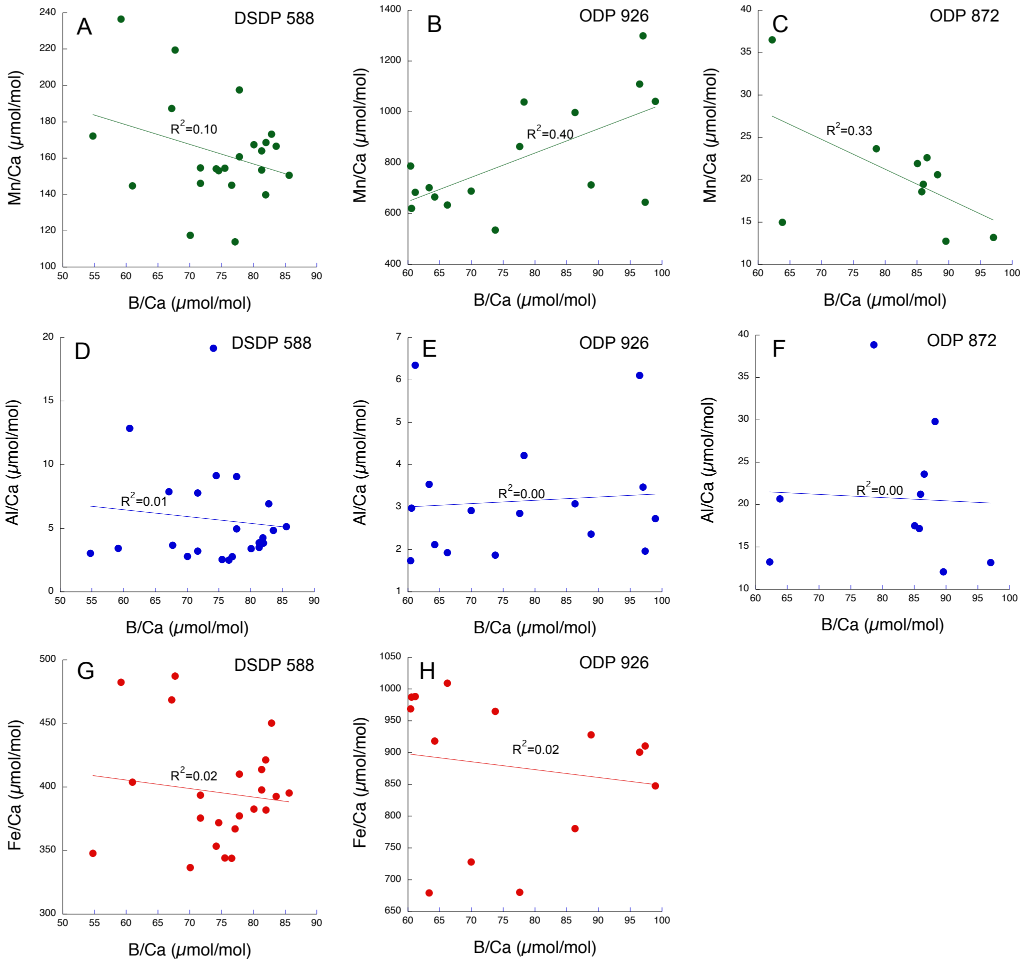


**Supplementary Figure 6** Miocene *T. trilobus* cross-plots of B/Ca and (A-C) Mn/Ca, (D-F) Fe/Ca and (D)Al/Ca ratios from DSDP site 588, ODP site 926, and ODP site 872 respectively.

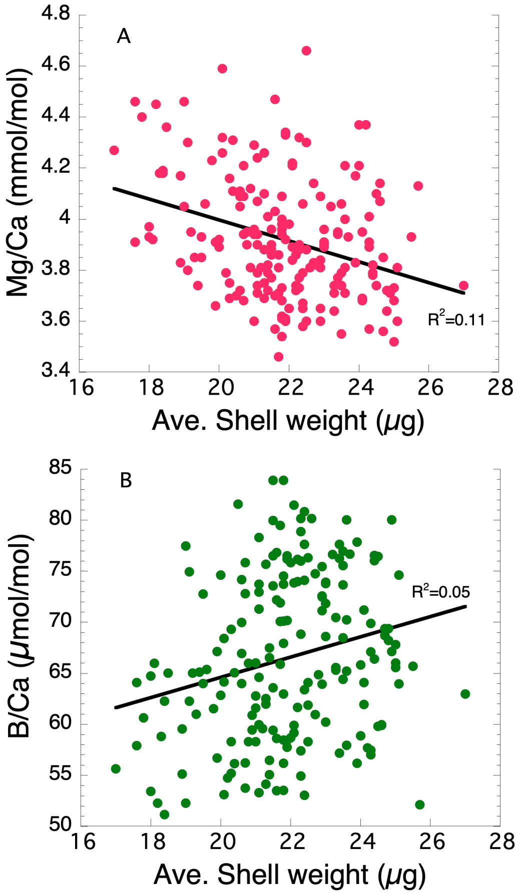


**Supplementary Figure 7** Cross-plots of *T. trilobus* (A) Mg/Ca mmol/mol and (B) B/Ca μmol/mol versus normalized shell weight from ODP site 761.

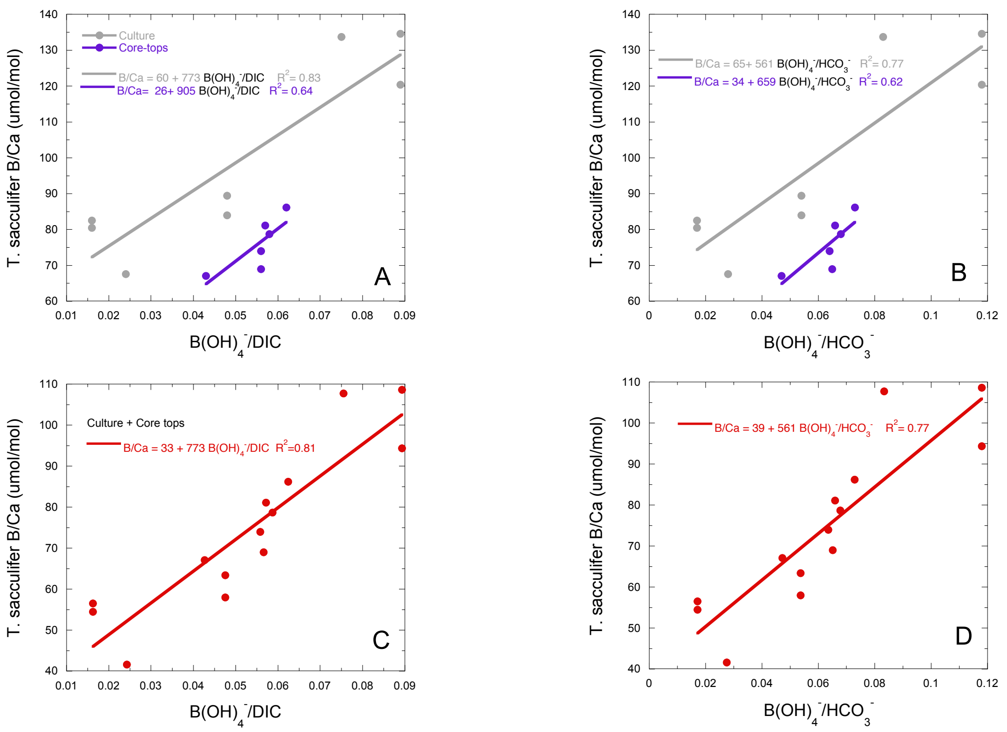


**Supplementary Figure 8** *T. sacculifer* B/Ca μmol/mol core-top and culture data plotted versus seawater (A) [$B(OH)_4^-$/DIC] and (B) [$B(OH)_4^-/HCO_3^-$]. B/Ca values are normalized to salinity of 35 using the culture derived B/Ca-salinity relationship for *T. sacculifer*[5]. Test size was adjusted using the core top derived B/Ca-test size relationship[6]. Ad hoc calibrations used in this study for (C) B/Ca-[$B(OH)_4^-$/DIC] and (D) B/Ca-[$B(OH)_4^-/HCO_3^-$] are based on the slope derived from culture relationship (Allen et al., 2012) and intercept from combined culture and core-top data[5,7].

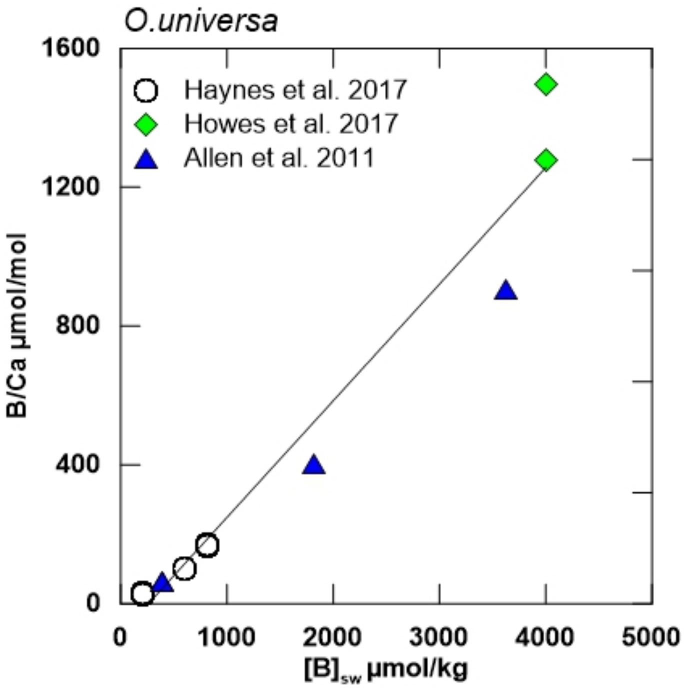


**Supplementary Figure 9** *O.universa* B/Ca µmol/mol culture experiment data with varying $[B]_{sw}$ µmol/kg. B/Ca data are normalized to salinity of 33 using the culture derived B/Ca-salinity relationship for *O.universa*[8]. B/Ca data are normalized to a DIC of 2000µmol/kg using the culture derived B/Ca-DIC relationship for *O.universa*[9].

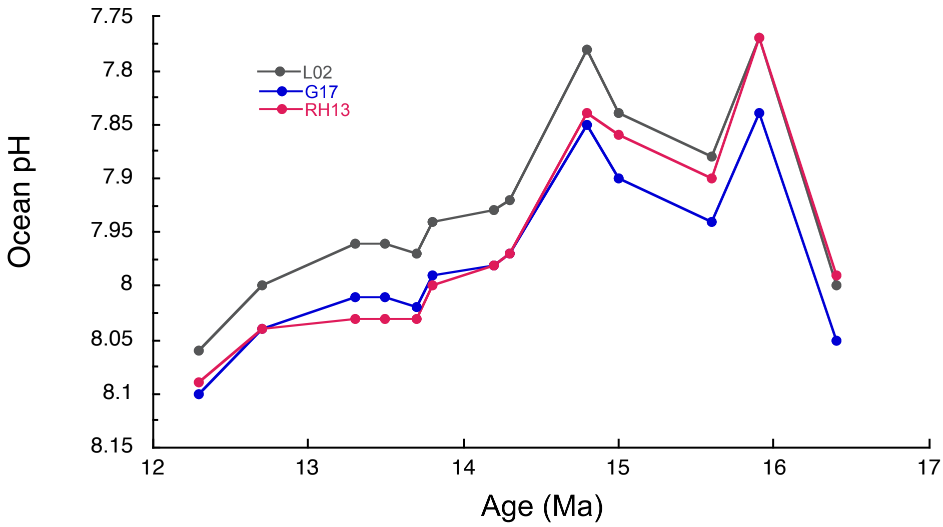


**Supplementary Figure 10** Median surface ocean pH estimates for ODP site 761 derived using boron isotope estimates and three $\delta^{11}B_{sw}$ scenarios[3] (L02; RH13; G17)

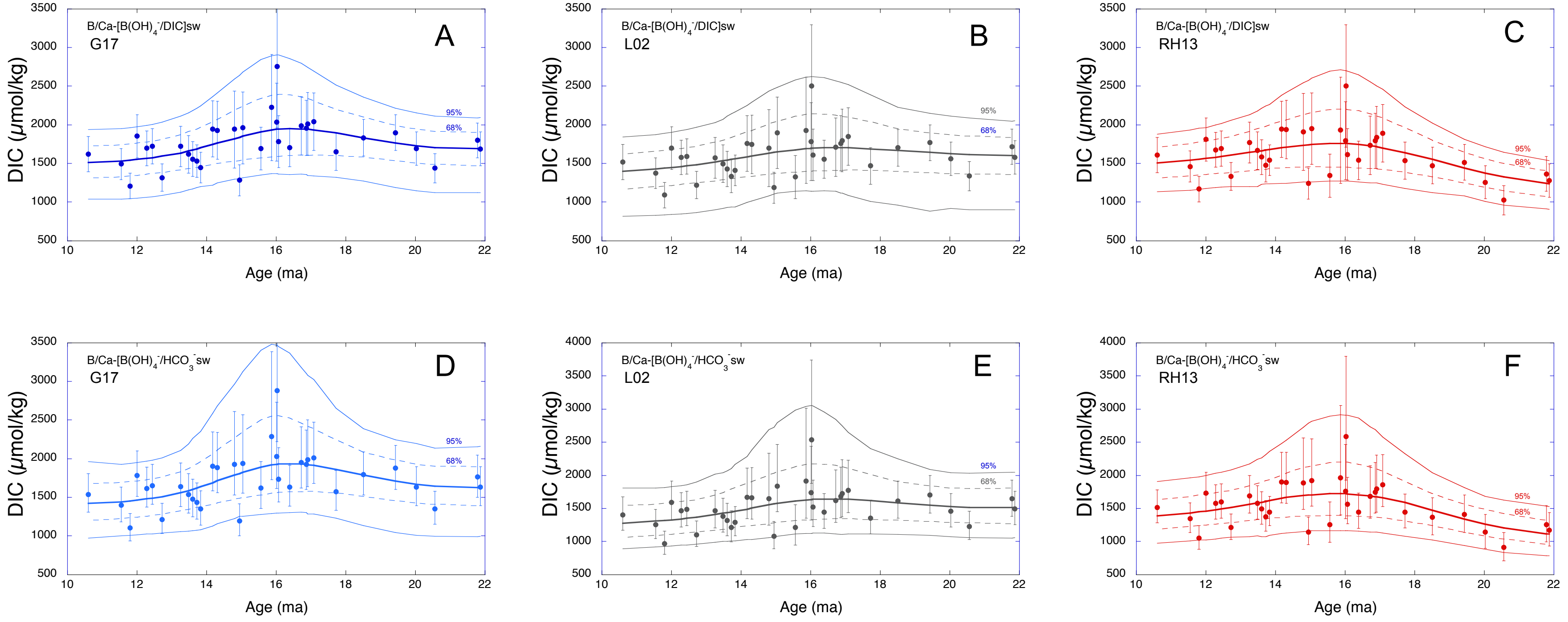


**Supplementary Figure 11** Uncertainty in the DIC estimates derived from B/Ca-$[B(OH)_4^-/DIC]_{sw}$ and $[B(OH)_4^-/HCO_3^-]_{sw}$ calibrations using a Monte Carlo approach for (A, D) G17, (B, E) L02, and (C, F) RH13 $\delta^{11}B_{sw}$ scenarios[3]. The error bars for each data point are 68% confidence and the blue line is a smoothed spline fit through the data and the blue bands are 68% and 95% around the smoothed fit line.

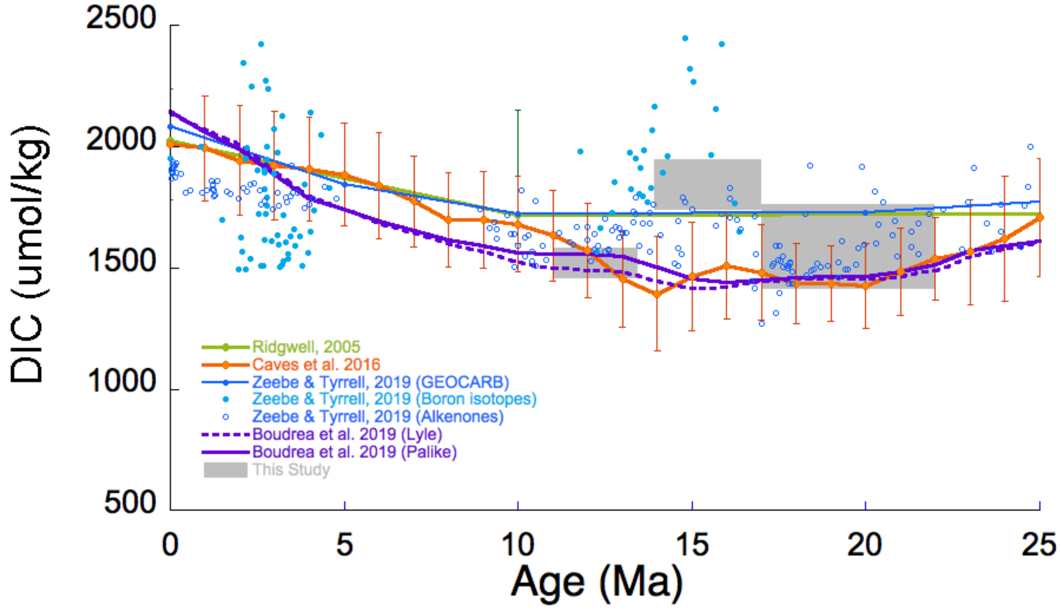


**Supplementary Figure 12** The range of DIC estimates derived from B/Ca-[$B(OH)_4^-$/DIC]$_{sw}$ calibration and three $\delta^{11}B_{sw}$ scenarios (grey shaded boxes) from the early Miocene (17-22.0 Ma), MCO (14-17.0 Ma) and post-MMCT (11.0-13.5 Ma) in comparison to DIC derived from geochemical modelling studies [10, 11, 12. 13]. Note the overlap in range of DIC estimates from this study with other approaches.

## Supplementary Tables

**Supplementary Table 1** Correlation between B/Ca and Mn/Ca in binned windows across the Middle Miocene

| Time interval (Ma) | $R^2$ |
|---|---|
| 11.5 to 12 | 0.30 |
| 12 to 12.5 | 0.01 |
| 12.5 to 13 | 0.05 |
| 13 to 13.5 | 0.04 |
| 13.5 to 14 | 0.29 |
| 14 to 14.5 | 0.01 |
| 14.5 to 15 | 0.03 |
| 15 to 15.5 | 0.15 |
| 15.5 to 16 | 0.01 |
| 16 to 16.5 | 0.20 |

**Supplementary Table 2** Summary of mean *T. trilobus* Sr/Ca values[14] during the Middle Miocene.

| Time Interval (Ma) | Average Sr/Ca (mmol/mol) |
|---|---|
| 12 to 13 | 1.11 |
| 13 to 14 | 1.14 |
| 14 to 15 | 1.13 |
| 15 to 16 | 1.13 |

**Supplementary Table 3** Summary of mean surface DIC (µmol/kg) concentrations during the early Miocene, MCO, and MMCT estimated using B/Ca-$[B(OH)_4^-/DIC]_{sw}$ *ad hoc* calibration.

| Time interval | | G17 | | | L02 | | | RH13 | | |
|---|---|---|---|---|---|---|---|---|---|---|
| | | **DIC (µmol/kg)** | **L68%** | **U68%** | **DIC (µmol/kg)** | **L68 %** | **U68%** | **DIC (µmol/kg)** | **L68 %** | **U68%** |
| Early Miocene | (17.0 to 22.0 Ma) | 1755 | 235 | 240 | 1623 | 257 | 259 | 1418 | 195 | 216 |
| MCO | (14 to 17 Ma) | 1944 | 346 | 420 | 1733 | 323 | 427 | 1792 | 319 | 431 |
| MMCT | (13.5 to 11.0 Ma) | 1579 | 215 | 222 | 1454 | 230 | 240 | 1573 | 214 | 238 |

**Supplementary Table 4** Summary of mean surface DIC (µmol/kg) concentrations during the early Miocene, MCO, and MMCT estimated using B/Ca-$[B(OH)_4^-/HCO_3^-]_{sw}$ *ad hoc* calibration.

| Time interval | | G17 | | | L02 | | | RH13 | | |
|---|---|---|---|---|---|---|---|---|---|---|
| | | **DIC (µmol/kg)** | **L68%** | **U68%** | **DIC (µmol/kg)** | **L68%** | **U68%** | **DIC (µmol/kg)** | **L68%** | **U68%** |
| Early Miocene | (17.0 to 22.0 Ma) | 1704 | 256 | 296 | 1533 | 252 | 310 | 1319 | 199 | 244 |
| MCO | (14 to 17 Ma) | 1921 | 361 | 564 | 1662 | 322 | 519 | 1752 | 334 | 461 |
| MMCT | (13.5 to 11.0 Ma) | 1493 | 226 | 260 | 1339 | 223 | 278 | 1472 | 219 | 264 |

**Supplementary Table 5** Change in DIC across the Miocene using B/Ca-[B(OH)$_4^-$/DIC]$_{sw}$ and B/Ca-[B(OH)$_4^-$/HCO$_3^-$]$_{sw}$ *ad hoc* calibrations.

| | **ΔDIC B/Ca-[B(OH)$_4^-$/DIC]$_{sw}$** | | | **ΔDIC B/Ca-[B(OH)4-/HCO3-]sw** | | |
|---|---|---|---|---|---|---|
| | G17 | L02 | RH13 | G17 | L02 | RH13 |
| Early Miocene to MCO | 189 | 110 | 374 | 217 | 129 | 434 |
| MCO to MMCT | 366 | 279 | 219 | 428 | 323 | 280 |

**Supplementary References**


1. Takahashi, T., S.C. Sutherland and A. Kozyr (2017). Global Ocean Surface Water Partial Pressure of $CO_2$ Database: Measurements Performed During 1957-2016 (LDEO Database Version 2016) (NCEI Accession 0160492). Version 3.3. NOAA National Centers for Environmental Information.Dataset.
2. Key, R. M. *et al.* A global ocean carbon climatology: Results from Global Data Analysis Project (GLODAP). *Global Biogeochemical Cycles* 18, doi:10.1029/2004gb002247 (2004).
3. Sosdian, S. M. *et al.* Constraining the evolution of Neogene ocean carbonate chemistry using the boron isotope pH proxy. *Earth and Planetary Science Letters* **498**, 362-376, doi:10.1016/j.epsl.2018.06.017 (2018).
4. Badger, M. P. S. *et al.* $CO_2$ drawdown following the middle Miocene expansion of the Antarctic Ice Sheet. *Paleoceanography* **28**, doi:10.1002/palo.20015 (2013).
5. Allen, K. A., Honisch, B., Eggins, S. M. & Rosenthal, Y. Environmental controls on B/Ca in calcite tests of the tropical planktic foraminifer species Globigerinoides ruber and Globigerinoides sacculifer. *Earth and Planetary Science Letters* **351**, 270-280, doi:10.1016/j.epsl.2012.07.004 (2012).
6. Ni, Y. Y. *et al.* A core top assessment of proxies for the ocean carbonate system in surface-dwelling foraminifers. *Paleoceanography* **22**, doi:10.1029/2006pa001337 (2007).
7. Foster, G. L. Seawater pH, $pCO_2$ and $CO_3^{2-}$ variations in the Caribbean Sea over the last 130 kyr: A boron isotope and B/Ca study of planktic forminifera. *Earth and Planetary Science Letters* **271**, 254-266, doi:10.1016/j.epsl.2008.04.015 (2008).
8. Allen, K. A. *et al.* Controls on boron incorporation in cultured tests of the planktic foraminifer Orbulina universa. *Earth and Planetary Science Letters* **309**, 291-301, doi:10.1016/j.epsl.2011.07.010 (2011).
9. Haynes, L. L. *et al.* Calibration of the B/Ca proxy in the planktic foraminifer Orbulina universa to Paleocene seawater conditions. *Paleoceanography* **32**, 580-599, doi:10.1002/2016pa003069 (2017).
10. Ridgwell, A. A Mid Mesozoic Revolution in the regulation of ocean chemistry. *Marine Geology* **217**, 339-357, doi:10.1016/j.margeo.2004.10.036 (2005).
11. Caves, J. K., Jost, A. B., Lau, K. V. & Maher, K. Cenozoic carbon cycle imbalances and a variable weathering feedback. *Earth and Planetary Science Letters* **450**, 152-163, doi:10.1016/j.epsl.2016.06.035 (2016)
12. Zeebe, R. & Tyrrell, T. History of carbonate ion concentration over the last 100 million years II: Revised calculations and new data. *Geochim. Cosmochim. Acta*, (2019)
13. Boudreau, B. P., Middelburg, J. J., Sluijs, A. & van der Ploeg, R. Secular variations in the carbonate chemistry of the oceans over the Cenozoic. *Earth and Planetary Science Letters* **512**, 194-206, doi:10.1016/j.epsl.2019.02.004 (2019).
14. Sosdian, S. *T. trilobus* Sr/Ca ratios from ODP 761 (Cardiff University, 2019).
